# A Natural History of Actinic Keratosis and Cutaneous Squamous Cell Carcinoma Microbiomes

**DOI:** 10.1128/mBio.01432-18

**Published:** 2018-10-09

**Authors:** David L. A. Wood, Nancy Lachner, Jean-Marie Tan, Stephanie Tang, Nicola Angel, Antonia Laino, Richard Linedale, Kim-Anh Lê Cao, Mark Morrison, Ian H. Frazer, H. Peter Soyer, Philip Hugenholtz

**Affiliations:** a Australian Centre for Ecogenomics, School of Chemistry and Molecular Biosciences, The University of Queensland, Brisbane, Queensland, Australia; b Dermatology Research Centre, The University of Queensland, Brisbane, Queensland, Australia; c Dermatology Department, Princess Alexandra Hospital, Brisbane, Queensland, Australia; d Diamantina Institute, The University of Queensland, Brisbane, Queensland, Australia; e Translational Research Institute, The University of Queensland, Brisbane, Queensland, Australia; f School of Mathematics and Statistics, Melbourne Integrative Genomics, The University of Melbourne, Victoria, Australia; University of Maryland, School of Medicine; University of Sydney; European Molecular Biology Laboratory

**Keywords:** 16S RNA, actinic keratosis, *Malassezia*, microbiome, skin, squamous cell carcinoma, *Staphylococcus aureus*

## Abstract

Cutaneous squamous cell carcinoma (SCC) is the second-most-common cancer in Australia. The majority of SCCs progress from premalignant actinic keratosis (AK) lesions that form on chronically sun-exposed skin. The role of skin microbiota in this progression is not well understood; therefore, we performed a longitudinal microbiome analysis of AKs and SCCs using a cohort of 13 SCC-prone immunocompetent men. The majority of variability in microbial profiles was attributable to subject, followed by time and lesion type. *Propionibacterium* and *Malassezia* organisms were relatively more abundant in nonlesional photodamaged skin than in AKs and SCCs. *Staphylococcus* was most commonly associated with lesional skin, in particular, sequences most closely related to Staphylococcus aureus. Of 11 S. aureus-like operational taxonomic units (OTUs), six were significantly associated with SCC lesions across seven subjects, suggesting their specific involvement with AK-to-SCC progression. If a causative link exists between certain S. aureus-like OTUs and SCC etiology, therapeutic approaches specifically targeting these bacteria could be used to reduce SCC.

## Abstract (precis)

Actinic keratosis (AK) and cutaneous squamous cell carcinoma (SCC) are two of the most common dermatologic conditions in Western countries and cause substantial morbidity worldwide. The role of human papillomaviruses under these conditions has been well studied yet remains inconclusive. One PCR-based study has investigated bacteria in the etiology of these conditions; however, no study has investigated the microbiomes of AK and SCC more broadly. We longitudinally profiled the microbiomes of 112 AK lesions, profiled cross sections of 32 spontaneously arising SCC lesions, and compared these to matching nonlesional photodamaged control skin sites. We identified commonly occurring strains of *Propionibacterium* and *Malassezia* at higher relative abundances on nonlesional skin than in AK and SCC lesions, and strains of Staphylococcus aureus were relatively more abundant in lesional than nonlesional skin. These findings may aid in the prevention of SCC.

## INTRODUCTION

Actinic keratosis (AK), also known as solar keratosis, is a condition where premalignant lesions develop on photodamaged skin. Risk factors for AK include chronic sun exposure, advancing age, fair skin, and immunosuppression (1, 2). AK lesions are very common in Western countries, being the most common dermatologic diagnosis in individuals over 45 years of age in North America (3), and occur on 60% of all men aged over 40 in Australia (4). If untreated, the lesions can naturally regress, remain stable, or progress to cutaneous squamous cell carcinoma (SCC), a form of keratinocyte carcinoma. In individuals with no history of keratinocyte carcinoma, each year a very small percentage (<0.075%) of AK lesions progress to SCC, dramatically increasing (up to 0.53%) in individuals with a history of keratinocyte carcinoma (5). While mortality from SCC is low (5), it is one of the most common cancers worldwide, and treatment of AKs and SCCs is a substantial and rising health care burden (4).

Human skin hosts resident microbial communities, including viruses, fungi, and bacteria, which may play a role in the etiology of SCC. The best studied of these are the human papillomaviruses (HPV), some of which have been shown to transform epithelial cells by genomic insertion and induce failures in cell cycle checkpoints in cervical, head, and neck SCCs (6, 7). While SCCs often contain HPV DNA, evidence for viral activity remains inconclusive (8). Human commensal yeasts from the genus *Malassezia* have been hypothesized as potential triggers of basal cell carcinoma (BCC) because they produce potent ligands targeting the carcinogenesis-associated aryl-hydrocarbon receptor and because there is an overlap of *Malassezia*-preferred body sites with locations where BCCs commonly form (9). There have been no reports associating *Malassezia* with SCCs. To our knowledge, only one study has investigated skin bacteria in relation to AK and cutaneous SCC, finding an enrichment of Staphylococcus aureus on the skin of patients with AK and SCC relative to its level on the skin of healthy patient-matched controls (10). Here, we more broadly explore the microbiomes of AK and SCC lesions and photodamaged nonlesional controls from the extensor surfaces of the forearms of an immunocompetent cohort with a history of SCC. Most microbiome variation was attributable to study subject; however, *Malassezia* and *Propionibacterium* operational taxonomic units (OTUs) were associated with nonlesional skin, and a subset of Staphylococcus aureus OTUs and one S. epidermidis OTU were significantly associated with SCCs in seven subjects, suggesting a specific involvement in SCC etiology. These results may aid in the treatment of premalignant AK and in the prevention of SCC.

## RESULTS

### Study design and participants.

**FIG 1:**
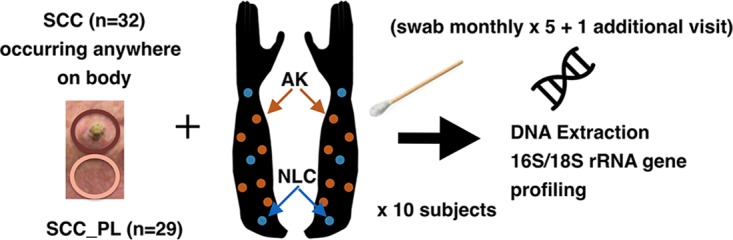
Sampling method used throughout the study. Up to six AK lesions were identified on each arm of each subject (orange points) and sampled once per month for 5 months and then once again no later than 9 months after the fifth visit. Sampling was performed by firmly applying and rotating a swab dipped in a sterile saline buffer to the skin site. For comparison to AKs, three nonlesional control (NLC) sites were selected on the photodamaged extensor surface of each forearm and spaced 10 cm apart, starting at the wrist and extending to the antecubital fossa. SCCs arising on any body site were also swabbed prior to excision, and the skin immediately adjacent to the SCC was swabbed for comparison (SCC_PL). Swab- and buffer-only controls were taken at each sampling session. Samples were briefly stored on ice in the clinic prior to –80°C storage in preparation for DNA extraction and rRNA gene amplicon profiling.

Ten men not known to be immunocompromised routinely presenting to the Dermatology Department in the Princess Alexandra Hospital, Brisbane, Australia, were recruited to a longitudinal study with informed consent (protocol HREC/11/QPAH/477; see Materials and Methods for inclusion criteria). Current and previous medical history, incidence of keratinocyte carcinoma, age, amount of melanin (skin phototype), and the number of current AKs were recorded during the first consultation (Table 1), and up to 6 AKs on each forearm (with a maximum of 12 in total) were selected for longitudinal monitoring throughout the study. For reliable identification, isolated lesions located between the wrist and elbow and distributed across the whole dorsal forearm were selected, and their locations were marked on sterile transparencies to facilitate reidentification at subsequent visits. Participants were sampled monthly for 5 months, and a subset of lesions was resampled once prospectively no later than 9 months after the fifth visit (Fig. 1). On each visit, AKs and the forearm were photographed, and current medications, topical-cream application, and time of last wash were recorded (Table S1a). AKs were evaluated for stability (i.e., they were stable, they had progressed to intraepidermal carcinoma or SCC, or they had regressed). No monitored lesion progressed to SCC, and 12 of 112 partially or fully regressed between the first and fifth visits (Table 1).

**TABLE 1:** Clinical metadata for all subjects in the study^*a*^

Subject	No. of SCCs	No. of BCCs	No. of AKs on left forearm	No. of AKs on right forearm	No. that regressed (partially regressed)	No. of SCCs sampled in this study
MS001	5–20	5–20	11–30	11–30	0	1
MS002	5–20	1–4	30+	30+	3	2
MS004	1–4	0	11–30	11–30	1 (1)	1
MS005	5–20	5–20	<10	11–30	3	1
MS006	1–4	0	30+	30+	2	3
MS007	1–4	5–20	30+	30+	1	1
MS008	5–20	5–20	30+	30+	0	6
MS009	5–20	1–4	30+	30+	0	1
MS010	5–20	5–20	30+	30+	1	5
MS011	20+	1–4	11–30	<10	0	8
MS012	1–4	1–4				1
MS013	1–4	0				1
MS014	5–20	1–4				6

a All subjects were immunocompetent men with skin phototype I (pale white skin) and II (fair skin). Mean age at study commencement was 68.3 years (range, 53 to 86 years). AK, actinic keratosis; SCC, cutaneous squamous cell carcinoma; BCC, basal cell carcinoma. Numbers of SCC and BCC refer to total lifetime incidences of keratinocyte carcinoma (formerly called nonmelanoma skin cancer). Numbers of AK (left and right forearms) refer to the number of AKs at study commencement. “No. that regressed” refers to the number of lesions that regressed from AK during the study. No lesions progressed to SCC. The number of SCCs sampled in this study is the number of spontaneously arising SCCs at any body site for which microbiome profiles were obtained.

10.1128/mBio.01432-18.2 TABLE S1 Contains supplemental Tables S1a to S1o. Download Table S1, XLSX file, 5.8 MB. Copyright © 2018 Wood et al.. 2018 Wood et al. This content is distributed under the terms of the Creative Commons Attribution 4.0 International license.

Three photodamaged skin sites on the extensor surface of each forearm spanning the wrist to elbow at 10-cm intervals were selected as nonlesional control (NLC) sites for comparison to the AK lesions (Fig. 1; Fig. S1a). NLC sites were standardized such that they were in the same relative position on the forearm across all participants. Due to the anticipated low rate of AK progression to SCC throughout the study, we additionally performed cross-sectional sampling of SCCs that developed at unmonitored locations on the longitudinally monitored subjects and supplemented these samples with SCC samples from three additional subjects recruited into the study (subjects MS012, MS013, and MS014) (Table 1). A single perilesional site located 3 cm from the SCC was also sampled to provide a region-specific control for each SCC (SCC_PL), as microbial community composition on human skin is site specific (11).

10.1128/mBio.01432-18.1 FIG S1 Contains supplemental Fig. S1a to S1v. Download FIG S1, PDF file, 2.3 MB. Copyright © 2018 Wood et al.. 2018 Wood et al. This content is distributed under the terms of the Creative Commons Attribution 4.0 International license.

Each AK, SCC, SCC_PL, and NLC site was sampled with a sterile cotton swab dipped in saline solution and firmly applied and rotated on the lesion in an area ∼1.5 cm in diameter (Fig. 1; see also Materials and Methods). Because skin swabbing results in low sample biomass, at each sampling session, one or more swabs were processed as normal without skin swabbing, resulting in 29 technical swab controls. Because 80% of the human population carries Staphylococcus aureus in the anterior nares either persistently (20%) or intermittently (60%) (12) and because endogenous S. aureus strains are known to cause infections within the carrier (13) and have been associated with SCC previously (10), the nasal mucosa was also swabbed at each visit to determine carrier status.

### Microbiome profiles.

DNA was extracted from each swab sample and PCR amplified using primers that broadly target bacterial, archaeal, and eukaryotic small-subunit rRNA genes (14). These primers enabled profiling of both prokaryotes and eukaryotes in the samples. Amplicons were pooled and sequenced, and they clustered into 99% identity threshold operational taxonomic units (OTUs). Putative chimeras were computationally identified, verified by manual inspection of alignments, and removed from subsequent analyses (18 in total). The fraction of human 18S rRNA gene sequences in these profiles was tolerably low (median fraction, 0.21; mean, 0.26) (Fig. S1b). Following human 18S rRNA removal, any OTU more frequently observed in the control swab profiles than in the AK profiles was excluded, which removed 235 OTUs consisting of probable reagent or sequencing contaminants (22% of all OTUs) (Fig. S1b; Table S1b). The remaining microbial read counts for skin samples ranged between 5,104 and 100,500 (mean, 18,420) (see Materials and Methods for full bioinformatic processing details). In total, microbial profiles were successfully obtained from 499 AK, 289 NLC, 44 nasal, 32 SCC, and 29 SCC_PL samples (Table S1c). The results for nine samples sequenced five times across the five sequencing runs used in the study were technically reproducible, indicating no batch effect (Fig. S1c). We observed a significantly higher proportion of human reads in the AK than in the NLC samples (Fig. S1d) but no significant difference between sample types for microbial read counts. We also observed variability in the proportion of human reads across subjects (Fig. S1d). These differences are likely due to variability in degrees of keratosis and therefore increased skin shedding between subjects and between the AK and NLC skin states.

### Actinic keratosis longitudinal study.

**FIG 2:**
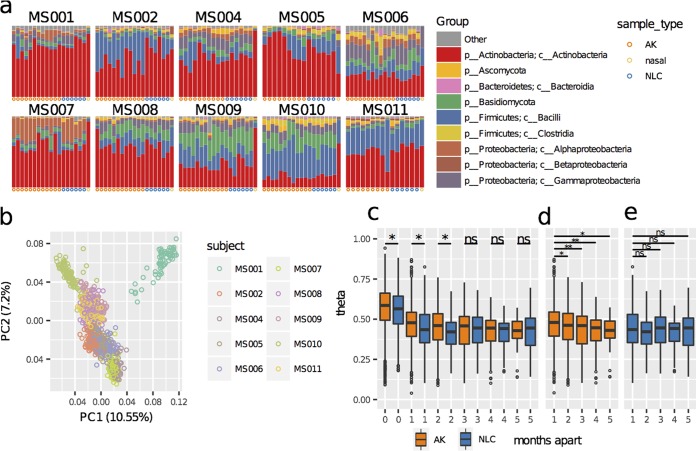
Differences in skin microbiome profiles across subjects and time. (a) Bar plots of microbial relative abundances for the seven most abundant bacterial classes (prefixed with c) and the two most abundant fungal phyla (prefixed with p) aggregated across five monthly time points for each lesion (AK, orange dots at the base of each plot) and for photodamaged nonlesional controls (NLCs) from the extensor surface of the lower arm (blue dots) and nasal samples (mustard yellow dots). (b) PCA ordination indicates that samples separate primarily by subject. PC1 and PC2, principal components 1 and 2, respectively. (c) Community differences between months measured using the Yue-Clayton theta distance (18). As theta values approach 1.00, the communities are more similar. “months apart” indicates the similarity of the skin microbiome profiles for the same lesion over time, taken from 1 to 5 months apart. Profiles at 0 months apart are microbiome profiles of separate AK and NLC samples taken at the same time as a frame of reference for temporal differences. AK community profiles were more similar to those of other AKs on the subjects’ arms during the same time point than they were to the profiles from the same AK across time. AK community profiles were on average significantly more stable than NLC profiles in the first 3 months; however, the magnitude of difference was small. ns, not significantly different. (d) AK-only theta distances indicate a small but significant decrease in AK community similarity over time. (e) NLC community similarity did not significantly decrease over the same period.

Our study design enabled microbial profile comparisons between lesions and photodamaged nonlesional skin on the same subject, between subjects, and longitudinally, since AKs can progress and regress over time. Permutational multivariate analysis of variance (PERMANOVA) across all AKs and NLCs (*n* = 788) indicated that 42.1% of between-sample variation was attributable to subject alone (permutation *F*-test significance = 0.001) (Table S1d). Bar plots of bacterial (class-level) and fungal (phylum-level) OTU groupings for each subject showed distinct interindividual variation of skin communities (Fig. 2a), supported by principal-component analysis (PCA) indicating strong clustering of samples by subject (Fig. 2b), which is consistent with previous reports of individualized human skin microbial composition (15–17). The combined factors of time and subject explained 16.9% of variance (permutation *F*-test significance = 0.001), and across all subjects, time alone explained 3.7% of variance (permutation *F*-test significance = 0.001), possibly influenced by seasonal changes.

To explore changes in community composition over time, the Yue-Clayton theta diversity index (18) was calculated between all samples. Across all subjects, community profiles were more similar between different forearm locations during the same visit than between identical locations across months, indicating that changes in the skin microbiomes of individuals occur across the entire forearm over time (Fig. 2c). With samples taken between 1 and 3 months apart, the mean theta indexes between AK communities were more similar than those between NLC communities; however, this significantly decreased to match NLC community similarity by the fifth month (Fig. 2d and e), suggesting that, on average, AKs have more-stable microbiomes than photodamaged nonlesional skin over short time frames but are no more stable over longer time frames. However, several subjects showed distinct differences in the levels of similarity of their skin microbiomes (Fig. S1e). For example, the microbial profiles of subject MS001 were distinct between the first visit and all other time points. Higher variability in AK community profiles was observed between visits than with NLC sample profiles from subject MS002, whereas both AK and NLC community profiles were relatively uniform over time in subjects MS004 and MS008, highlighting not only individualized microbiomes but individualized patterns of change in microbiomes.

Variation attributable to sample type (AK or NLC) for each subject explained 0.7% of variance (permutation *F*-test significance = 0.001), and across all subjects, sample type explained 0.1% of total variance (permutation *F*-test significance = 0.001). Ordination constrained by sample type also produced a significant separation of AK and NLC samples (redundancy analysis permutation test *P* = 0.01) (Fig. S1f). These results indicate a small but significant difference between the microbiomes of AK and photodamaged nonlesional skin, with the majority of variance attributable to subject and time.

**FIG 3:**
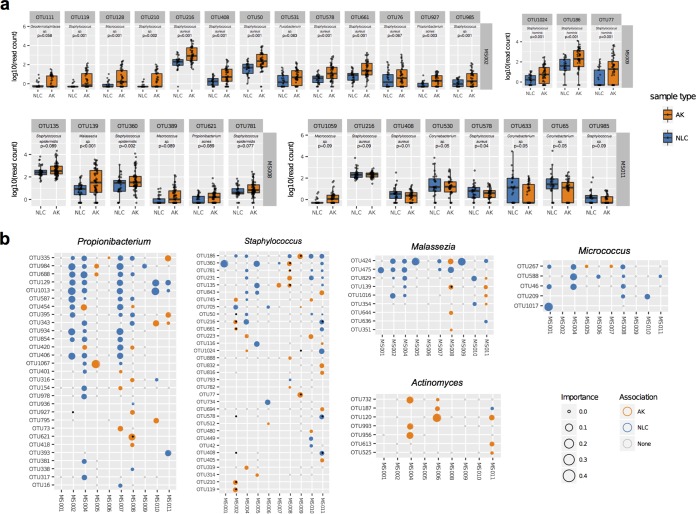
OTU associations with AK and NLC samples. (a) Boxplots showing the log_10_ read counts for 30 differentially abundant OTUs (adjusted log ratio test *P* value < 0.1), which were calculated with DESeq2 between AKs and NLCs for each subject. *Staphylococcus* OTUs were encountered frequently and were frequently higher in relative abundance in AK samples than in other samples. (b) Multivariate analysis using sPLS-DA identified sets of OTUs that maximally discriminate between AK and NLC samples for each subject. An OTU’s contribution to variation separating samples on both components of the model was used to determine sample type association. This method identified 707 OTUs associated with either AKs or NLCs across all subjects. Shown here are all OTUs associated with a sample type from five selected genera. Each circle represents an OTU tested in each subject. The color represents the sample type association, and the size of the circle is proportional to the sPLS-DA “importance” metric. Small open gray circles indicate that the OTU was testable in the subject but that no sample type association was identified. If no circle is present, the OTU did not have sufficient reads in that sample for testing. Black dots indicate that the OTU was differentially abundant in the univariate analysis. *Propionibacterium* was significantly enriched in NLC-associated OTUs (adjusted *P* value, 1.02e^–8^, chi-square test). *Malassezia* and *Micrococcus* were also enriched in NLC-associated OTUs; however, differences between these genera did not reach significance. OTUs highlighted with black dots were also identified as differentially abundant in the DESeq2 univariate analysis.

Given the strong intersubject variability, identification of key OTUs associated with sample type (AK or NLC) was performed for each subject. We applied two types of methods, one univariate (DESeq2 [19]) and the second multivariate (sparse partial least-squares discriminant analysis [sPLS-DA] [20]; see Materials and Methods). Collectively, 30 OTUs were differentially abundant according to univariate testing (likelihood ratio test, all adjusted *P* values < 0.1); of these, including 15 OTUs belonging to the genus *Staphylococcus* found on subjects MS002 (9 OTUs, by phylogenetic tree placement closest to S. aureus or undetermined), MS008 (3 OTUs, all closest to S. epidermidis), and MS009 (3 OTUs, all closest to S. hominis) (Fig. 3a and Table S1e), the majority were present more often in AK samples than in other samples. Three S. aureus OTUs and one undetermined *Staphylococcus* OTU on subject MS011 were higher on nonlesional control skin than in AK samples. The 11 non-*Staphylococcus* OTUs that significantly associated with AK samples were also subject specific and belong to the bacterial genera *Corynebacterium* (3 OTUs), *Macrococcus* (3 OTUs), *Propionibacterium* (2 OTUs), *Fusobacterium* (1 OTU), and *Geodermatophilus* (1 OTU) and the fungal genus *Malassezia* (1 OTU). These results indicate that OTUs associated with AK were subject specific and were predominantly from the genus *Staphylococcus* and a limited number of other bacterial and fungal taxa.

Microbiome data are sparse, overdispersed, and compositional, and these attributes can affect the sensitivity of parametric statistical methods (21). Therefore, in addition to univariate analysis, the multivariate method sPLS-DA was used to identify sets of OTUs that maximally discriminate between AK and NLC samples for each subject. Sparse PLS-DA is similar to principal-component regression analysis; however, the method fits a linear regression model to best explain the variance between samples when the *Y* variable is categorical (in this case, either the AK or the NLC sample type). Application of sPLS-DA to samples from individual time points for each subject indicated in many cases that a signature of OTUs that separated AK and NLC samples in the first two principal components could be selected (Fig. S1g). For additional statistical power, repeated measurements for each sample across the six longitudinal visits were analyzed using a multilevel decomposition approach that normalizes across time points and allows inclusion of the longitudinal samples as replicates (20). OTUs were then determined as associated with either AK or NLC sample type based on their contribution to the variation separating samples on both components of the model, referred to as their “importance.” In total, across all subjects, 707 OTUs (394 unique) were identified as associated with a sample type (307 AK and 398 NLC samples) (Table S1f). Corroborating the univariate results, 19 of the 30 differentially abundant OTUs were correctly identified as AK or NLC associated in the multivariate analysis (indicated with black points in Fig. 3b), and the remaining 11 were not identified as associated with either sample type, likely due to sPLS-DA seeking a linear combination of OTUs, as opposed to considering each OTU individually. Also consistent with the univariate analysis, subject-specific differences were apparent. For example, within subject MS005, three *Propionibacterium* OTUs most similar to Propionibacterium acnes were AK associated yet NLC associated in other subjects. Similarly, *Malassezia* OTU 424 was AK associated in subject MS008 yet NLC associated in other subjects. The skin microbiome of subject MS001 largely lacked *Staphylococcus* organisms and were dominated by actinobacteria, including *Arthrobacter*, *Brevibacterium, Propionibacterium*, and *Micrococcus*. In this subject, a *Micrococcus* OTU (1017) was strongly NLC associated; however, few distinguishing AK-associated OTUs were identified. The increased sensitivity afforded by the multivariate analysis produced sets of OTUs that discriminate between sample types for most subjects (Fig. S1h to S1q).

In addition to the multiple AK-associated OTUs, many were identified as NLC associated. Eleven *Propionibacterium* OTUs were associated with NLC samples in subject MS002, and of these, two similar to P. acnes (OTUs 129 and 1013) were identified as NLC associated in subjects MS004, MS007, MS0010, and MS0011 (Fig. 3b). Notably, *Malassezia* OTU 424 was NLC associated in six subjects (MS002, MS004, MS005, MS007, MS009, and MS0011), and another *Malassezia* OTU (475) was NLC associated in five subjects (MS001, MS002, MS004, MS007, and MS008). Collectively, these data suggest that there is a subset of NLC-associated OTUs that are important in the nonlesional skin microenvironment. In contrast, AK-associated OTUs tended to be subject specific.

Grouping OTUs at the genus level revealed variable results with regard to AK or NLC association (Table S1g). The genus *Propionibacterium* was significantly enriched for OTUs associated with nonlesional skin (adjusted *P* value of the chi-square test, 1.02e^–8^) (Fig. 3b). OTUs within *Malassezia* and *Micrococcus* were more commonly NLC associated; however, neither of these genera reached significance. Likewise, OTUs within *Actinomyces* were more frequently associated with AK than with NLC; however, the number did not reach significance. *Corynebacterium*, *Streptococcus*, and *Staphylococcus* were not significantly associated with either sample type at the genus level (Fig. 3b), although specific *Staphylococcus* OTUs were the most significantly differentially abundant of all OTUs in the univariate analysis (Fig. 3a).

**FIG 4:**
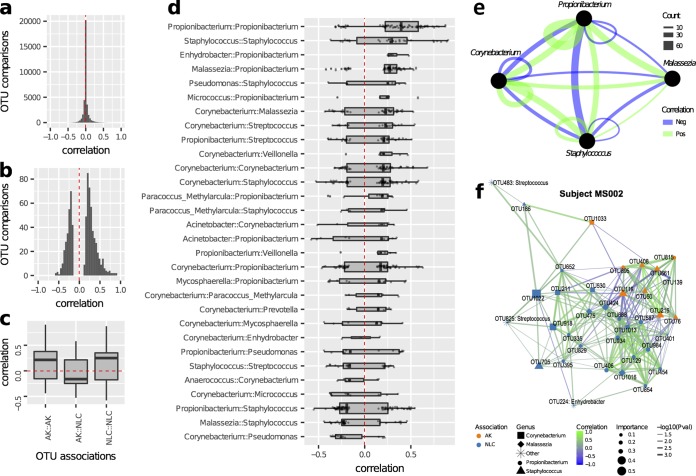
Abundance correlation network analysis of AK- and NLC-associated OTUs. (a) Histogram of all pairwise OTU comparisons noting that the vast majority of values are around zero and are not significantly correlated. (b) All significant correlations (*P* < 0.05) note a difference in *y* axis range from that of the previous panel. (c) Significant correlations involving two OTUs associated with the same sample type (AK::AK and NLC::NLC) were positive, whereas correlations involving OTUs from different sample types (AK::NLC) were generally negative (the red line indicates mean correlation across all pairwise OTUs). (d) Significant correlations by genus from most positively correlated to most negatively correlated. Intragenus OTU correlations were generally positively correlated (73 out of 97). (e) Correlation network graphically showing major inferred relationships between frequently observed genera. Correlations between *Malassezia* and *Propionibacterium* were generally positive, whereas correlations between *Staphylococcus* and *Propionibacterium* were generally negative, suggesting competitive exclusion between the latter two genera. (f) The network for subject MS002 shows two highly interconnected modules, one NLC associated (containing predominantly *Propionibacterium* and *Malassezia* OTUs) and one AK associated (containing predominantly *Staphylococcus* OTUs), that are negatively correlated with each other, suggesting a preference for lesional or nonlesional environments.

To understand the dynamics between OTUs and how microbial interactions might contribute to AK pathology, abundance correlation networks were calculated for each subject (see Materials and Methods). OTUs were included in the networks if they were associated with AK or NLC sample types from the sPLS-DA and were significantly positively or negatively correlated in abundance with another AK- or NLC-associated OTU (*P* value < 0.05, absolute correlation > 0.1). Across all subjects, 722 OTU comparisons were significant (Fig. 4a and b). Correlations between OTUs associated with the same sample type were significantly higher on average than correlations between different OTUs associated with different sample types (Fig. 4c and AK::NLC, two-tailed *t* test, *P* value = 9.08e^–17^). Relative abundances of OTUs from the same genus were generally positively correlated (73 out of 97), as were *Malassezia* and *Propionibacterium* OTUs and *Staphylococcus* and *Corynebacterium* OTUs (Fig. 4d and e). However, correlations between *Staphylococcus* and either *Propionibacterium* or *Malassezia* OTUs were generally negative. For example, the network for subject MS002 contains a cluster of AK-associated *Staphylococcus* OTUs, which negatively correlated with multiple *Propionibacterium* and *Malassezia* OTUs frequently associated with NLC samples in other subjects (Fig. 4f). These interactions suggest that not only do OTUs from these genera have a preferred skin microenvironment, they actively compete and/or are antagonistic toward each other in that preferred environment.

### Cutaneous squamous cell carcinoma cross-sectional study.

**FIG 5:**
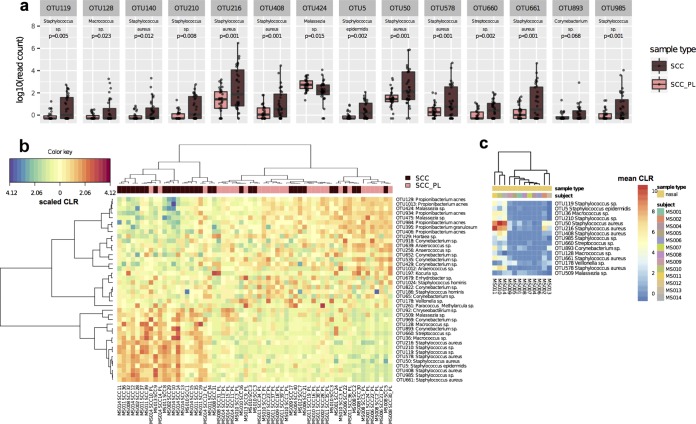
Identification of OTUs associated with SCC or perilesional skin (SCC_PL). (a) Fourteen OTUs found to be differentially abundant between SCC and SCC_PL samples according to univariate analysis, of which 13 were relatively more abundant in SCC microbiomes. (b) Multivariate analysis highlights a microbial signature differentiating SCC and SCC_PL samples. SCCs from subjects MS002, MS008, MS0011, MS0012, MS0013, and MS0014 were notable for their high relative abundances of several Staphylococcus aureus OTUs, whereas *Propionibacterium* and *Malassezia* OTUs were typically of higher relative abundance in SCC_PL samples. (c) Nasal microbiomes indicate potential carriage of several OTUs associated with SCCs, including a number of the S. aureus OTUs.

Cutaneous SCCs developing on any body site of longitudinally monitored subjects were swabbed prior to biopsy or excision and included with SCCs sampled from additional subjects for the cross-sectional study. Perilesional skin adjacent to the SCC (SCC_PL) was also sampled to provide a site-specific control. SCC and SCC_PL samples were obtained from a variety of body sites, including the head, chest, arms, and legs (Table S1h). In total, 32 SCCs and 29 SCC_PL samples were profiled. Univariate analysis with DESeq2 indicated that 13 OTUs were significantly more abundant in SCCs and that one *Malassezia* OTU was significantly more abundant in SCC_PL samples (adjusted *P* value < 0.1) (Fig. 5a; Fig. S1r; Table S1i). Of the 13 SCC-associated OTUs, 10 were members of the genus *Staphylococcus*, 6 of which were most similar to S. aureus, 1 of which was most similar to S. epidermidis, and 3 of which could not be resolved to the species level. Multivariate sPLS-DA aiming to discriminate the SCC and SCC_PL sample types resulted in a clear separation of samples into two groups, one consisting mostly of SCCs dominated by S. aureus OTUs, in particular OTUs 216 and 50 (also found by univariate analysis), and a second consisting mostly of the SCC_PL samples dominated by six *Propionibacterium* and two *Malassezia* OTUs (Fig. 5b and Table S1j). A weakly positive correlation was observed between the number of SCCs that developed on subjects throughout the study and the mean abundances of S. aureus OTUs 216 and 50 (Fig. S1s). SCCs from subjects MS002, MS008, MS0010, MS0011, MS0013, and MS0014 were enriched in S. aureus OTUs, whereas SCC microbiomes from the remaining subjects more closely resembled PL skin. Three subjects belonging to the S. aureus-dominated SCC group were persistent carriers of S. aureus OTUs in high relative abundances on their skin (both NLC and AK samples) and also in their nares, whereas most PL-like subjects were intermittent carriers and typically had much lower abundances (Fig. 5c; Fig. S1t and S1u), suggesting that S. aureus carriage alone may be an SCC risk factor. However, not all S. aureus OTUs identified in the study were significantly associated with SCC lesions, suggesting that SCC association is not a species-wide phenomenon. Two of the six *Propionibacterium* and both *Malassezia* OTUs that associated with SCC_PL samples in the multivariate analysis were among the most frequently associated with NLC samples in the AK longitudinal study (Table S1k), further confirming their prevalence in nonlesional skin. An abundance correlation network was calculated using SCC and SCC_PL samples across all subjects. This network revealed interactions and structures similar to those of the network from subject MS002 and suggests potential competitive exclusion between clusters of *Staphylococcus* OTUs and the cluster of *Propionibacterium* and *Malassezia* OTUs (Fig. S1v).

## DISCUSSION

In this study, we investigated the microbiomes of actinic keratosis (AK) and cutaneous squamous cell carcinoma (SCC) in immunocompetent men longitudinally and cross-sectionally. Operational taxonomic units (OTUs) belonging to the genera *Propionibacterium* and *Malassezia* were frequently higher in abundance in photodamaged nonlesional control (NLC) samples taken from the extensor surface of the lower arm than in both forearm AK samples and SCC samples from any body site. These taxa are among the most abundant and common skin-resident microbial genera. Both are lipophilic and share preferred sebaceous habitats (22). The most common *Propionibacterium* species on human skin, P. acnes, hydrolyses triglycerides found in sebum and adheres to the released fatty acids (23). Genome analysis of the most abundant *Malassezia* species on human skin, *Malassezia globosa*, indicates that it lacks the potential to synthesize fatty acids and therefore relies on obtaining these compounds from its environment (24). The observed cooccurrence of these two taxa (Fig. 4; Fig. S1v) is reflective of their shared ecological niche and likely complementary metabolic functions. Both genera have been heavily investigated in the context of skin disorders. While the presence of specific strains of P. acnes is associated with acne vulgaris (25), this species was found to be statistically associated with healthy skin control subjects in studies of psoriasis (26) and dandruff (27). *Malassezia* species are causative of skin disorders; however, the pathogenesis of these disorders is complex, and pathogeneses differ depending on species, strain, host genetics, environmental conditions (including body site and seasonal changes), and morphological state (28). AKs are characterized by a rough, scaly surface involving atypical keratinocytes in the lower epidermis, potentially reducing the availability of sebum, which may account for the observed decrease in the relative abundances of *Propionibacterium* and *Malassezia* species that rely on sebum for growth (24). A decrease of *Malassezia globosa* was also recently observed in atopic dermatitis samples (29). The loss of these taxa from AKs may exacerbate lesion severity by disrupting the microbiome homeostasis and further reducing moisture and increasing pH; conversely, recolonization may assist in lesion regression. P. acnes also produces antimicrobial compounds (30), and reduction of this taxon in AKs may provide an opportunity for other microorganisms to flourish.

*Staphylococcus* was the most notable genus associated with AKs and SCCs having pronounced differential abundances in both the longitudinal and cross-sectional studies (Fig. 3a and 4a; Tables S1e and S1i). *Staphylococcus* species are abundant commensals on human skin; their preferred body sites are areas high in humidity, such as the naval, inguinal crease, gluteal crease, feet, popliteal fossa, and antecubital fossa (11). While AK-associated *Staphylococcus* OTUs were largely subject specific, six S. aureus OTUs identified in the study were consistently associated with SCCs across seven subjects. The most abundant of these, S. aureus OTUs 50 and 216, were detectable on the skin and nares of almost all subjects (Fig. 4c; Fig. S1u). This is consistent with the PCR-based findings of Kullander and colleagues where S. aureus was enriched in SCC lesions relative to seborrhoeic keratosis lesions, basal cell carcinoma lesions, and non-lesional control samples from the same subjects (10). Furthermore, S. aureus is the most frequently isolated organism from burn scars, chronic wounds, and sites of osteomyelitis that progress into SCCs (31, 32). An estimated 15% of human cancers have an infectious source (33). Helicobacter pylori involvement in several types of gastric cancer has been known for decades and is the largest single cause of infection-related cancers (34). The etiology of carcinogenesis caused by bacterial pathogens (and also viral and noninfectious irritants) typically involves inflammation (reviewed in reference 35). Such a mechanism is possible with cutaneous SCC in that S. aureus infection initiates inflammation-related signaling and the release of cytokines such as tumor necrosis factor alpha (TNF-α), already identified as involved in SCC genesis (36). If particular strains of S. aureus contribute to SCC genesis, such strains may serve as biomarkers for SCC risk, and lesions with prolonged infection would be ideal candidates for monitoring and targeting interventional treatment. We further speculate that the high incidence of SCCs observed in some immunosuppressed patients might be linked to the inability of the immune system to keep specific microbial triggers of SCC in check.

## MATERIALS AND METHODS

### Study design.

Ten immunocompetent men were recruited to the longitudinal study, and informed consent was obtained (per protocol HREC/11/QPAH/477). Criteria for inclusion included male gender, three to six clinically visible and discrete AK lesions on each forearm, and a history of SCC. Exclusion criteria included oral antibiotic use in the preceding 3 months or current topical AK treatment. Men were chosen because of their higher AK and SCC incidence than among women (37, 38) and because of the potential variability introduced arising from differences in the skin microenvironments and microbial compositions between genders (39). Using known AK-to-SCC lesion progression rates (5), we expected that between three to six monitored lesions would progress to SCC during the study. Therefore, to ensure sufficient SCC samples, we recruited an additional three men who were not known to be immunocompromised (subjects MS012, MS013, and MS014), who presented to the dermatology clinic during routine check-ups, and who were found to have one or more SCC lesions. Inclusion and exclusion criteria for the cross-sectional subjects were the same as for the longitudinal subjects.

Each AK, NLC, SCC, and SCC_PL site was sampled with a sterile swab dipped in 0.15 M sodium chloride solution and firmly applied and rotated on the lesion in an area ∼1.5 cm in diameter (Fig. 1). Each sample was immediately placed on ice for up to 20 min in the clinic, and then all samples from the session were stored at −80°C until DNA extraction.

### DNA extraction and sequencing.

Genomic DNA was extracted using the PowerSoil DNA isolation kit (MO BIO), with slight modifications based on the work of Costello et al. (40). Specifically, an additional incubation step of 65°C for 10 min was included after addition of 60 µl buffer C1; the subsequent vortexing step was reduced to 5 min, and DNA was eluted into 50 µl of buffer C6. DNA concentrations were measured using the Qubit double-stranded DNA (dsDNA) high-sensitivity assay kit (ThermoFisher Scientific). Small-subunit ribosomal RNAs were PCR amplified using the broad-specificity primer pair 926F and 1392R (14), modified to contain Illumina-specific adapter sequences (926F, 5′-TCGTCGGCAGCGTCAGATGTGTATAAGAGACAG *AAACTYAAAKGAATTGRCGG-*3′, and 1392R, 5′-GTCTCGTGGGCTCGGGTCTCGTGGGCTCGGAGATGTGTATAAGAGACAG*ACGGGCGGTGWGTRC*-3′). Libraries were constructed according to the standard 16S Metagenomic Sequencing Library Preparation guide (27 November 2013 version) from Illumina, with the only modification being the use of Q5 Hot Start high-fidelity 2× master mix (New England Biolabs) as per the manufacturer’s protocol (with a thermocycler profile of denaturation at 98°C for 2 min and 25 cycles of 98°C for 10 s, 55°C for 30 s, and 72°C for 30 s, with a final extension at 72°C for 2 min). Resulting PCR amplicons were purified using Agencourt AMPure XP beads (Beckman Coulter) as per the manufacturer’s instructions. Purified amplicons were indexed with unique 8-bp barcodes using the Illumina Nextera XT 384 sample index kit A-D (Illumina; FC-131-1002). Indexed amplicons were pooled in equimolar concentrations and sequenced on the MiSeq sequencing system (Illumina) using paired-end sequencing with V3 300-bp chemistry according to the manufacturer’s protocol. A subset of nine samples (all right-arm AK samples) were resequenced on each of the sequencing plates to serve as technical controls.

### Sequence data processing.

The first 23 bases of each read was trimmed with Trimmomatic (41) v0.33 to remove primer sequences, and the distal end was quality trimmed using a sliding window of 4 bases and an average base quality above 15. All sequences were then hard trimmed to 250 bases to facilitate clustering, and any sequences shorter than 250 bases were excluded from further analysis. If samples were resequenced due to low read counts, sequences were pooled and analyzed as a single sample. Operational taxonomic units (OTUs) were clustered at 99% similarity using the QIIME (42) v1.8.0 pick_open_reference_otus.py workflow with the clustering tool uclust (43) (v1.2.22q) against a combined 16S and 18S rRNA gene reference data set of Greengenes (44) (version 2013/05) and SILVA (45) (version 119), respectively. The use of high-similarity OTUs (99%) was necessary in this study, as closely phylogenetically related OTUs were associated with different sample types and different subjects. Chimeric OTUs were detected using ChimeraSlayer (46) v2010-04-29 after sequences were aligned with pynast (47) v0.1, and alignments were inspected manually and removed from subsequent analyses. Taxonomy was assigned to representative OTU sequences using the BLASTn (48) (v2.4.0) top hit to the combined 16S and 18S rRNA reference database. All OTUs matching metazoan 18S rRNA (almost exclusively human), plant 18S rRNA, or plastids were removed. Any OTU with an abundance of less than 0.05% in all samples was also removed. The final OTU table is provided as Table S1o in the supplemental material.

### Data analysis and statistics.

All analyses were performed in R v3.2.3, and all R source codes are available upon request. Because skin swabbing results in low microbial biomass, multiple technical controls were obtained during sampling to enable removal of putative contaminants whose source was reagents, swabs, and technicians. Any OTU with mean higher relative abundance in the swab controls than in the AK samples was removed from the OTU table (Fig. S1b). After these putative contaminants were removed, any sample with less than 5,000 reads was deemed a failure and removed from analysis. Sample read depth spanned 2 orders of magnitude; therefore, prior to analysis, all samples were rarefied to a maximum of 20,000 reads using the rrarefy function in vegan v2.3-3 (49). Rarefaction removed reads from 229/893 noncontrol samples. Total OTU removal from these samples ranged between 1 and 82 (median, 10) or, proportionally, between 0.2% and 80.0% of reads (median, 33.2%).

In order to remove the compositional dependency of relative abundance data, prior to PCA and heatmap visualizations, OTU counts were transformed to centered-log-ratio (CLR) values (50). Heatmaps were created with the pheatmap function from pheatmap v1.0.8 (51), boxplots were created with the geom_boxplot function in the v2.2.1 ggplot2 package, principal-component analysis was performed with prcomp using the parameters center and scale set to true, and redundancy analysis was performed using the function rda in the vegan package (v2.3-3) (49) and default parameters. The proportion of variance explained by each factor (PERMANOVA) was calculated using the rarefied count data with the *adonis* function within the package vegan v2.3-3 (49). Permutations were set to 999, and no “strata” argument was used. Adding a stratum of subject resulted in negligible changes to significance and the percentage of explained variance for each factor.

Differentially abundant OTUs were identified with the univariate method DESeq2 (19) (v1.10.1). DESeq2 fits the data to a negative binomial distribution and then tests for significant differences for each OTU between groups using a generalized linear model. An OTU table for each subject was created comprising only OTUs with ≥10 reads in at least one sample. We applied a model with time (visit number) as a reduced factor to accommodate the longitudinal samples as replicates and tested using the log ratio test (LRT). For SCC-to-SCC_PL comparisons, OTUs were tested for differential abundances using the DESeq function with default parameters and no reduced model. In all cases, result tables were extracted with the results function, with both independent filtering and the Cooks cutoff set to false.

sPLS-DA was run as described in the mixOmics pipeline (20). As in the univariate analysis, each subject was processed separately. OTUs were excluded if they comprised less than 200 reads across all samples taken from a given subject. Repeated measurements for each sample across the five longitudinal visits were analyzed using a multilevel decomposition approach that normalizes across time points and allows inclusion of the longitudinal samples as replicates. The optimal number of OTUs was selected using the tune.splsda function, given a vector of values ranging from 5 to 100. Heatmaps were created from the CLR values using the mixOmics CIM method, with both the parameter scale and transpose set to true. Discrimination between SCC and SCC_PL sample types was performed as described above except without the repeated-measure design, as not all subjects had multiple SCCs.

Abundance correlation networks were calculated using FastSpar, an optimized version of the method SparCC (52) using default parameters (https://github.com/scwatts/fastspar). Correlations were considered if the significance was less than 0.05, and both OTUs were either AK or NLC associated in multivariate analysis, or in the case of the SCC networks, both OTUs were SCC or SCC_PL associated.

### Ethics.

This study was ethically approved under protocol number HREC/11/QPAH/477.

### Code availability.

All R scripts for analysis and shell scripts for data processing are available upon request.

### Data availability.

All raw sequencing data were deposited at SRA under BioProject ID PRJNA395624. Lesion metadata are available in Table S1l. Sample metadata are available in Table S1m. OTU taxonomy assignments are available in Table S1n. The count-based OTU table for all OTUs with a maximum relative abundance greater than 0.05% in any sample and sample and lesion metadata are available in Table S1o.
