# Supplementary material for: A Natural History of Actinic Keratosis and Cutaneous Squamous Cell Carcinoma Microbiomes
Source: mBio. 2018 Oct 9;9(5):e01432-18. doi: 10.1128/mBio.01432-18 (PMC6178618; doi:10.1128/mBio.01432-18)
Supplement: FIG S1 [file mbo005184106sf1.pdf]

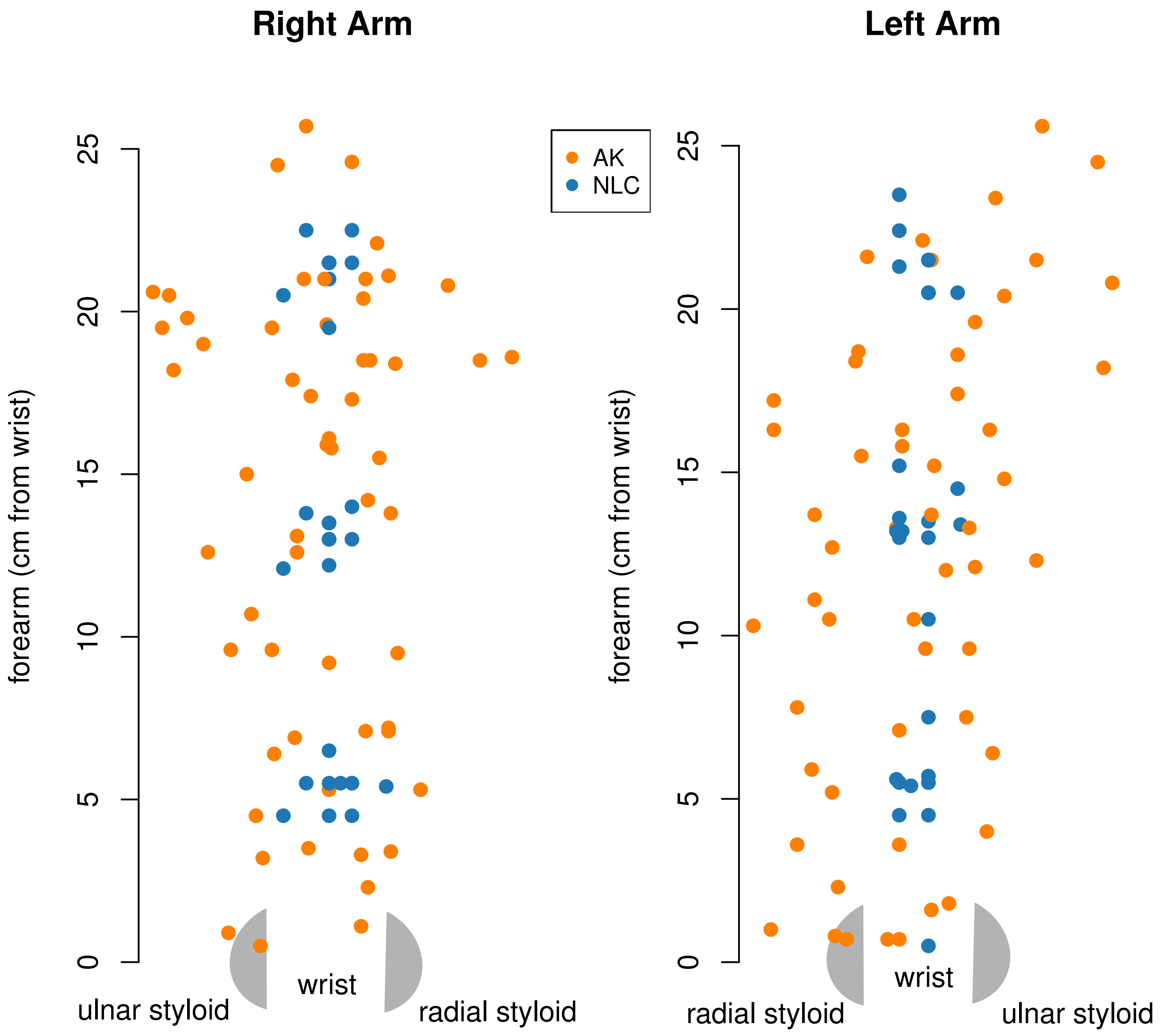

Figure S1a. Location of AK lesions and photo-damaged skin (non-lesional) controls (NLC) for each arm of all 10 longitudinal subjects. Lesion locations are relative to the centre of the wrist. NLC samples were chosen at 10 cm gaps such that they spanned the length of the arm and were not too close to any AK lesions on that subject.

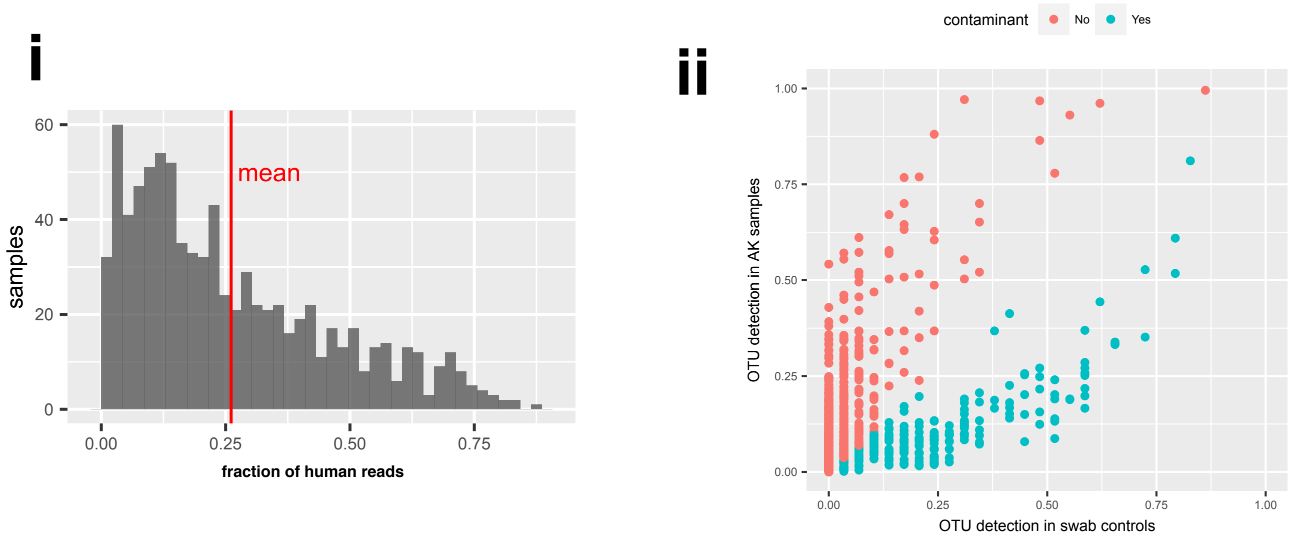

Figure S1b. Human 18S and putative microbial 16S contaminants. (i) Frequency of human 18S read fraction in each sample (mean 0.26), indicating that the total number of human reads was low enough to allow for suitable microbial profiling. (ii) A total of 29 swab controls were taken to provide negative controls for the low-biomass skin samples. Any OTU detected more frequently in these controls (red points), was removed from the analysis entirely.

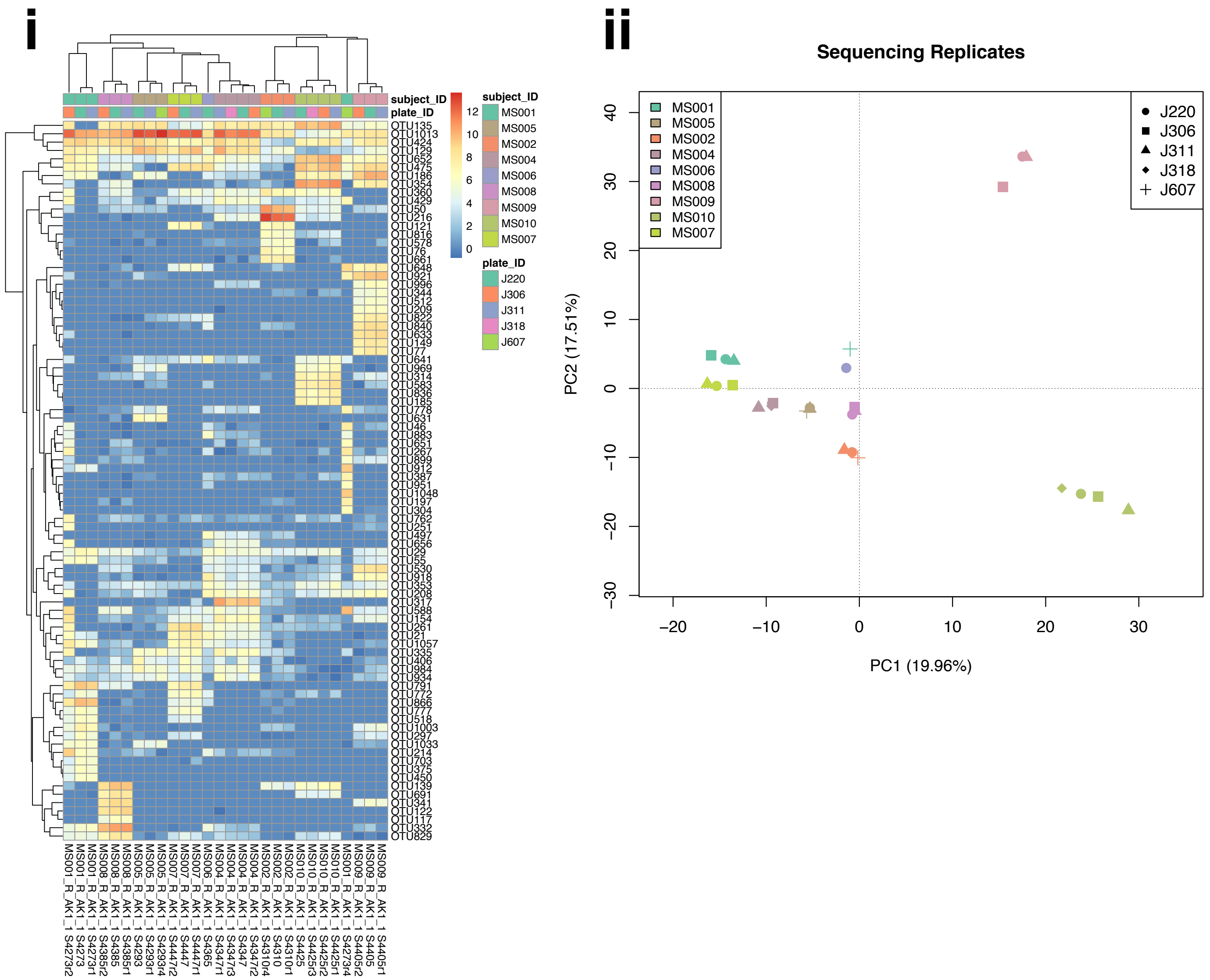

Figure S1c: Sequencing data reproducibility. (i) Heatmap showing abundance of any OTU with a CLR value >= 6 in any sample (columns) repeated across all five sequencing plates. (ii) Principal component analysis showing technical replicates for each sample and plate. Sequencing data were reproducible across plates.

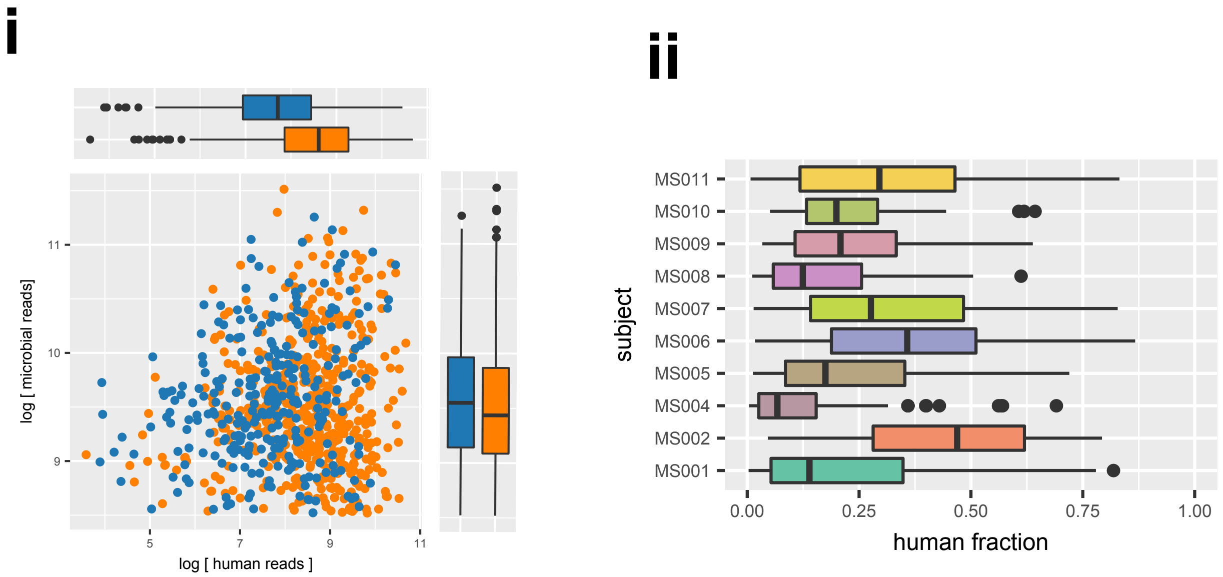

Figure S1d. Human and microbial read fraction. (i) Total number of human 18S and microbial 16S reads for both AK and NLC samples. AK samples had significantly higher numbers of human 18S reads. (ii) Variability of the fraction of human 18S reads in each sample for each subject.

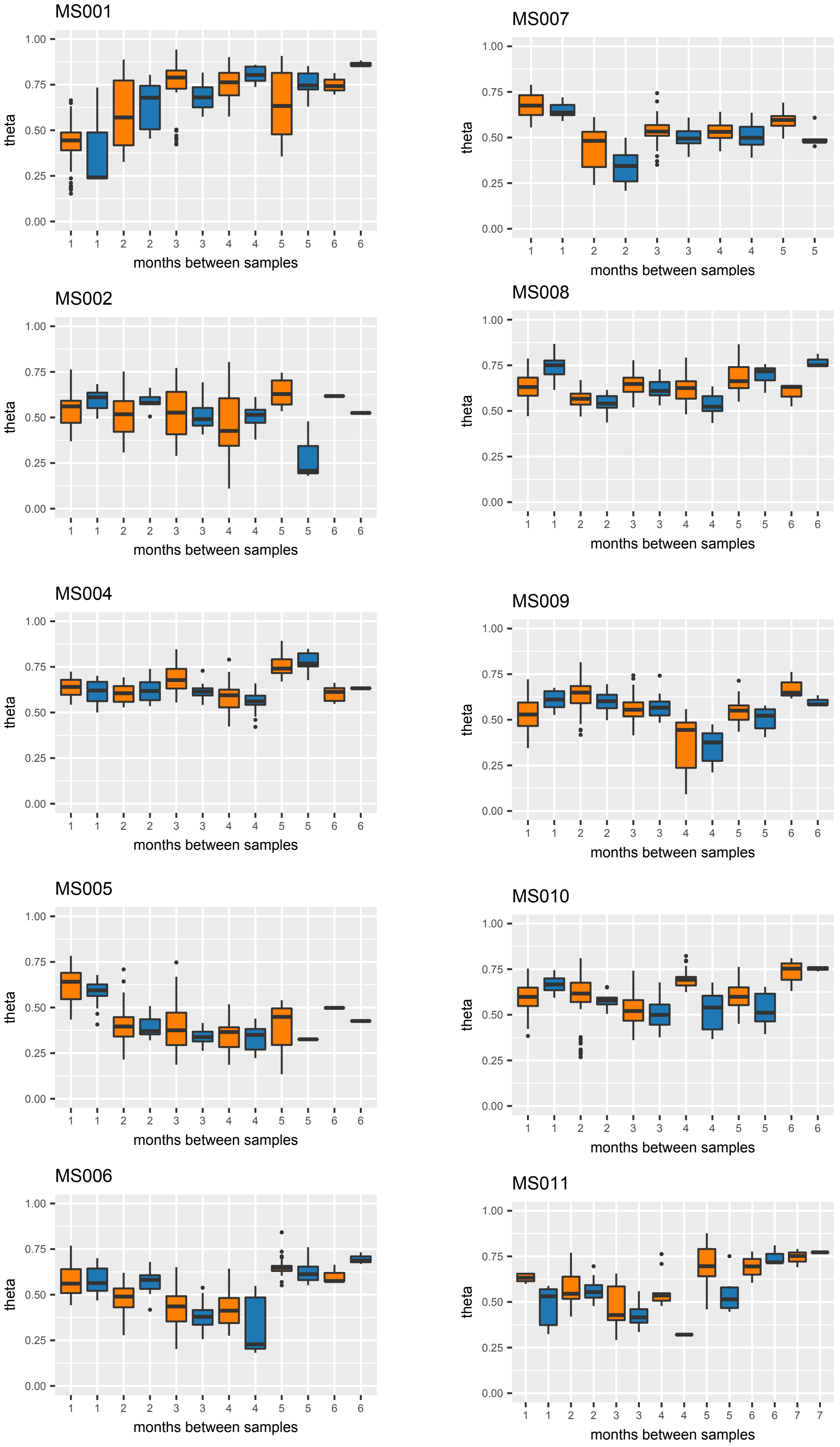

Figure S1e. Stability of AK and NLC microbiome communities over time. Yue-Clayton *theta* values were calculated for community comparisons (higher values are more similar). Data for each subject is shown in a single plot. Differences between the communities profiled at the same sampling site for actinic keratosis (AK, orange boxplots) and non-lesional photodamaged control samples (NLC, blue boxplots) are shown for each timepoint (months one to six). Some subjects have more stable microbiomes (eg MS008) than others (eg, MS005). Some timepoints are dissimilar to others (eg MS001, month 1). Differences between AK and NLC community similarity is observed in subject MS002.

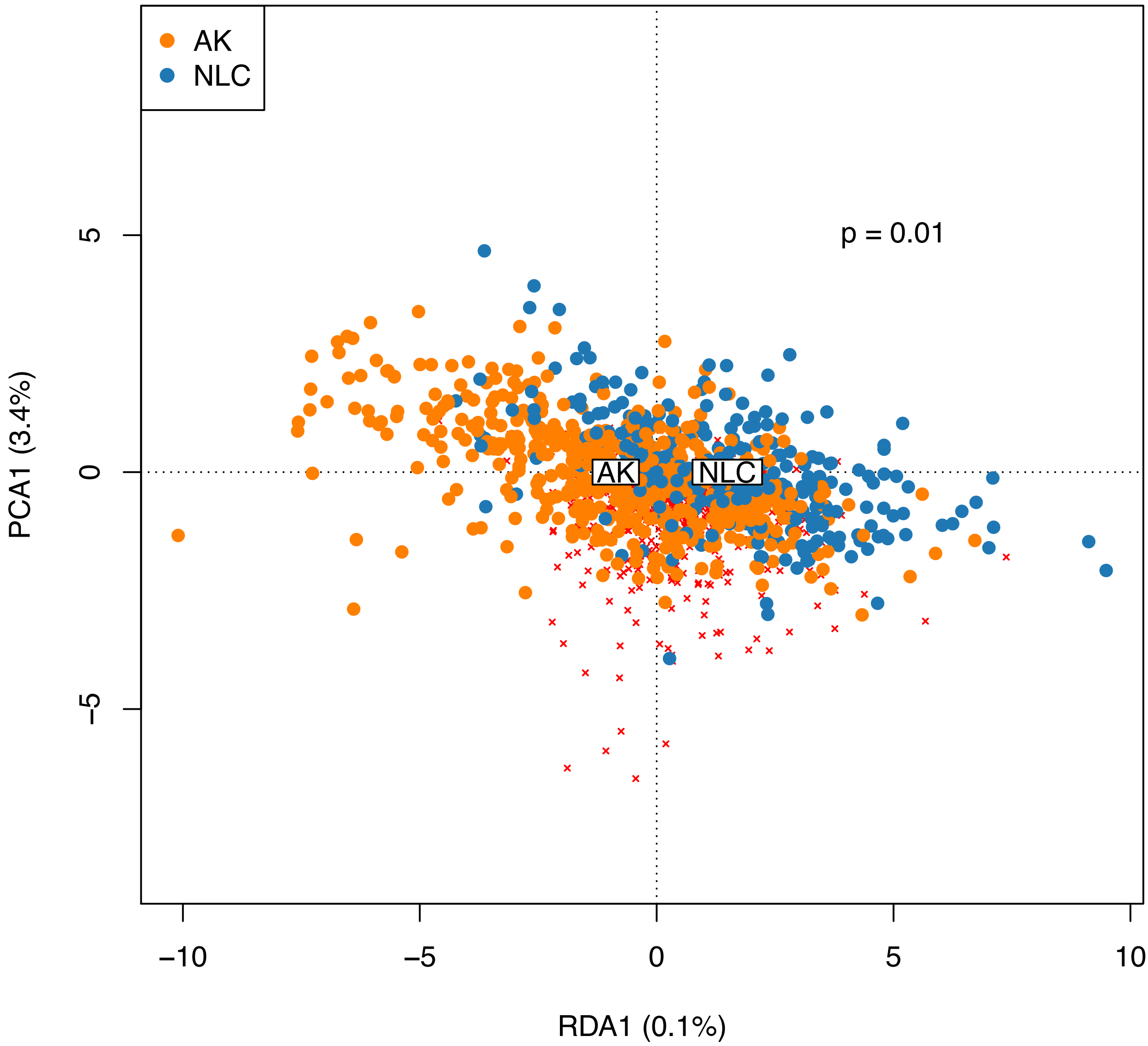

Figure S1f. Redundancy analysis separates AK and NLC sample types. Redundancy analysis of microbial profiles for all actinic keratosis (AK, orange points), and non-lesional photodamaged control (NLC) samples, constrained by sample type, demonstrates significant separation. Red crosses are operational taxonomic units (OTUs).

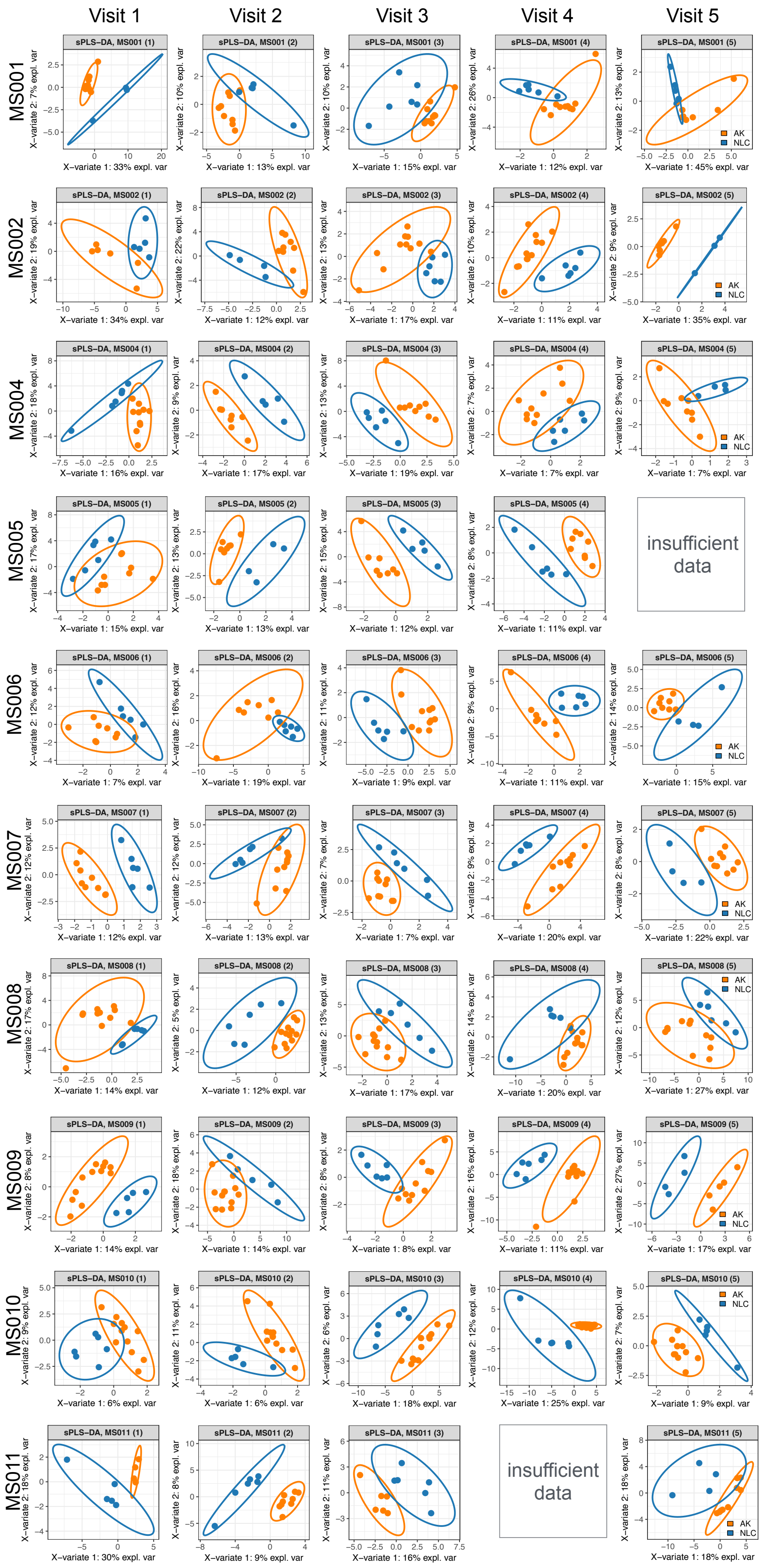

Figure S1g. sPLS-DA PCA plots of samples by time and subject. OTUs were selected with sPLS-DA that maximally discriminate between sample types (AK and NLC) for each subject and time point, and used to generate PCA plots. Separation between sample types was observable at individual time points.

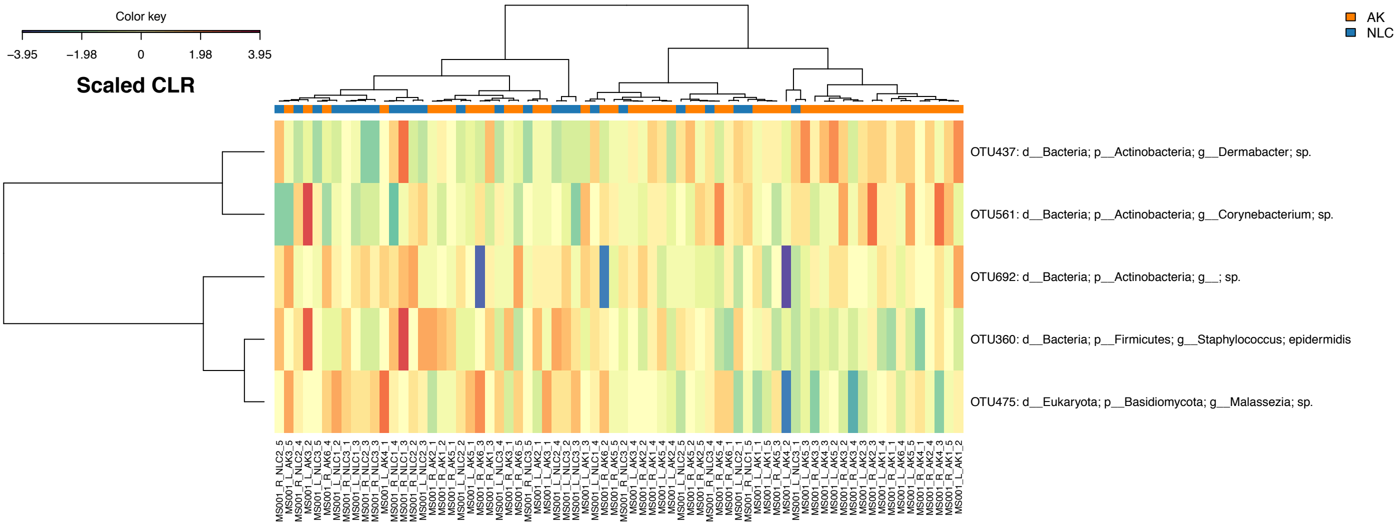

Figure S1h. Multivariate analysis highlight a microbial signature separating AK and NLC samples for subject MS001.

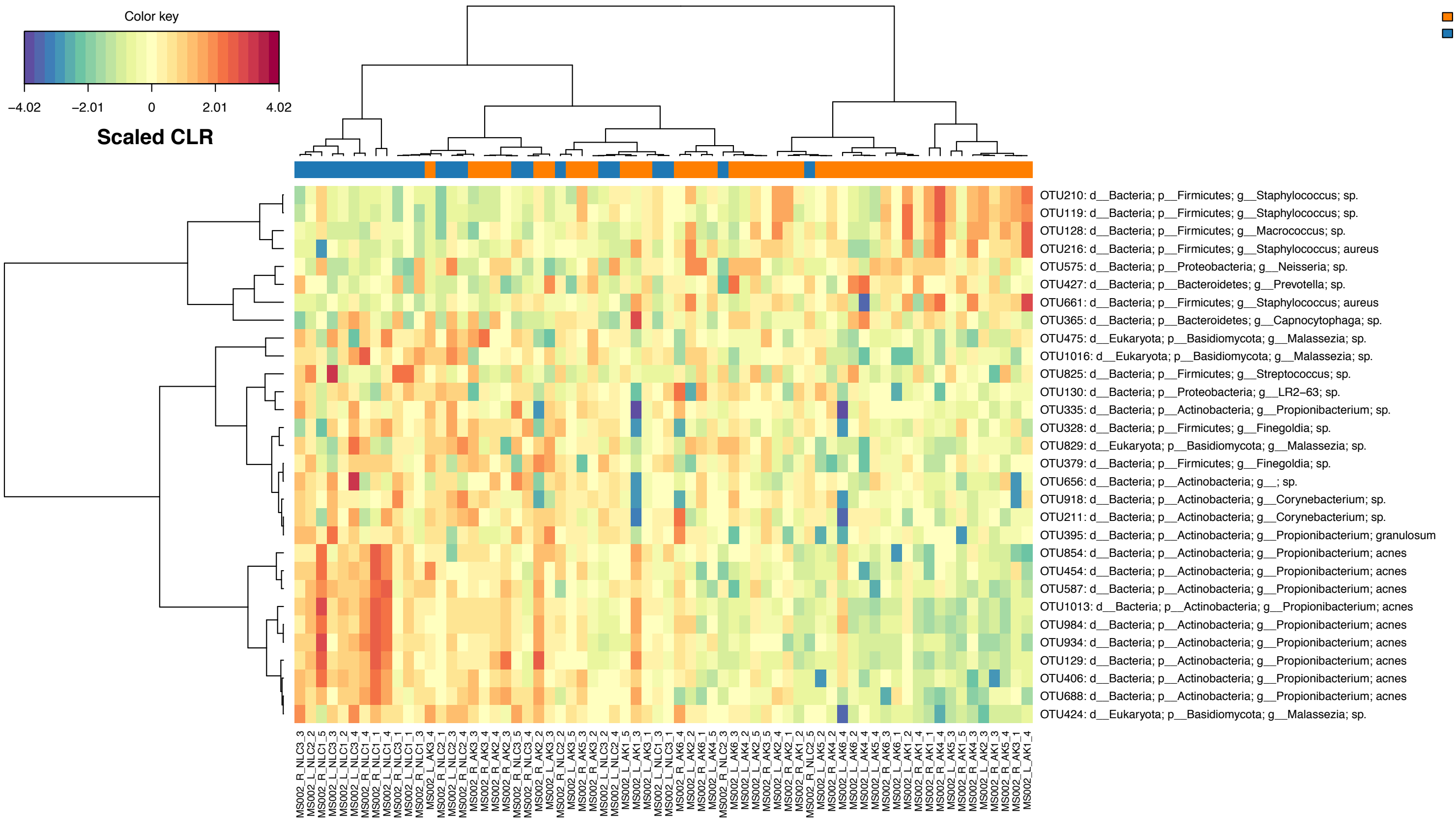

Figure S1i. Multivariate analysis highlight a microbial signature separating AK and NLC samples for subject MS002.

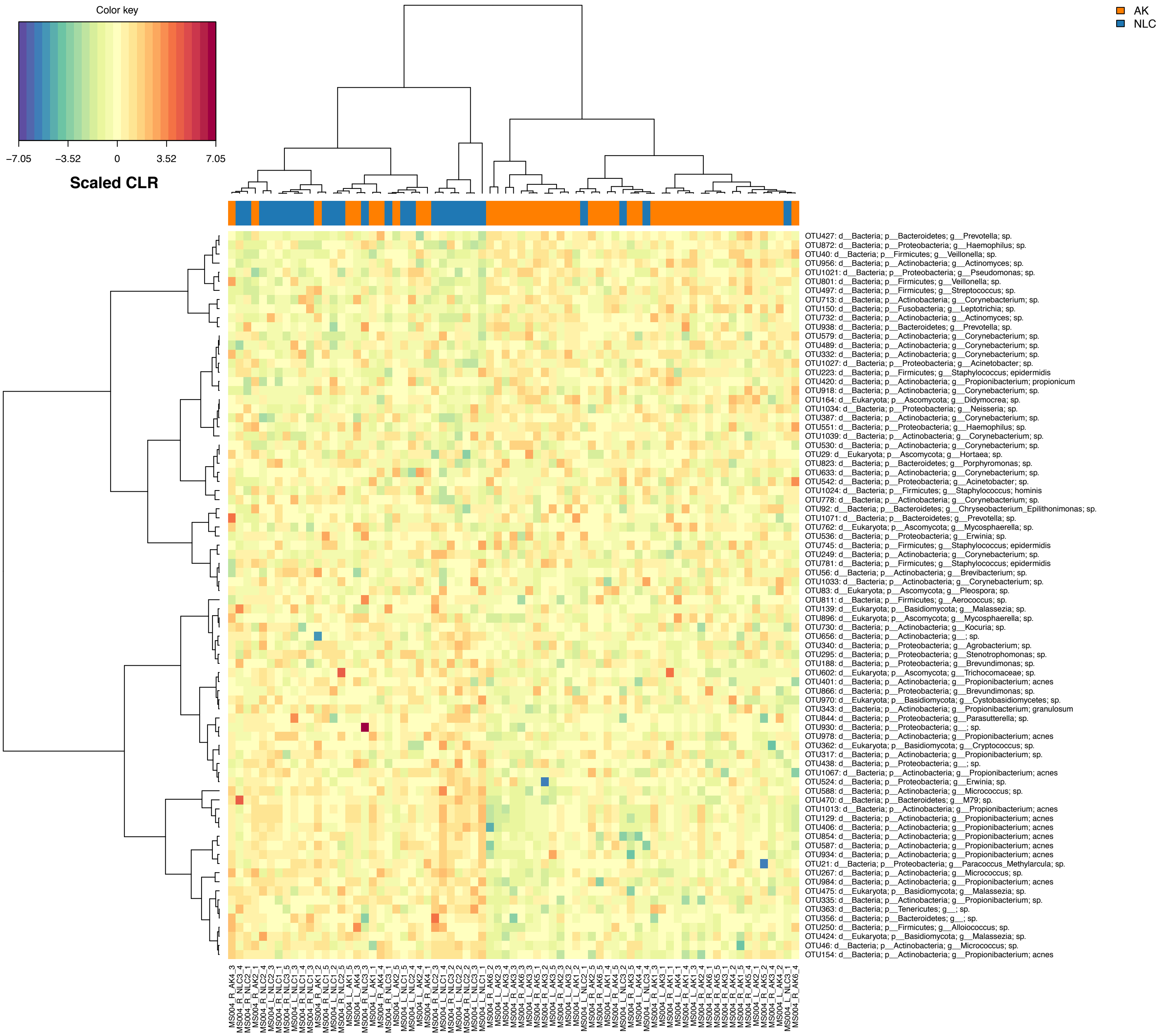

Figure S1j. Multivariate analysis highlight a microbial signature separating AK and NLC samples for subject MS004.

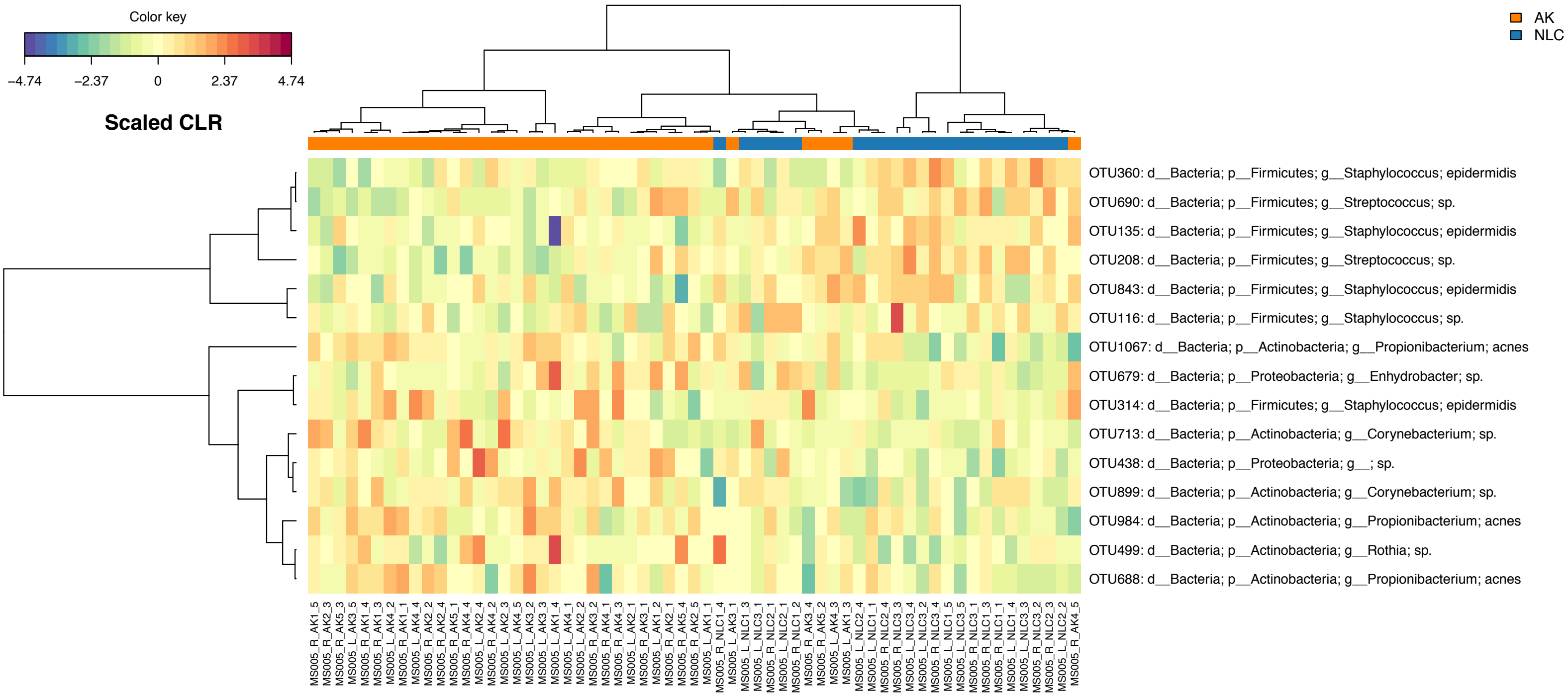

Figure S1k. Multivariate analysis highlight a microbial signature separating AK and NLC samples for subject MS005.

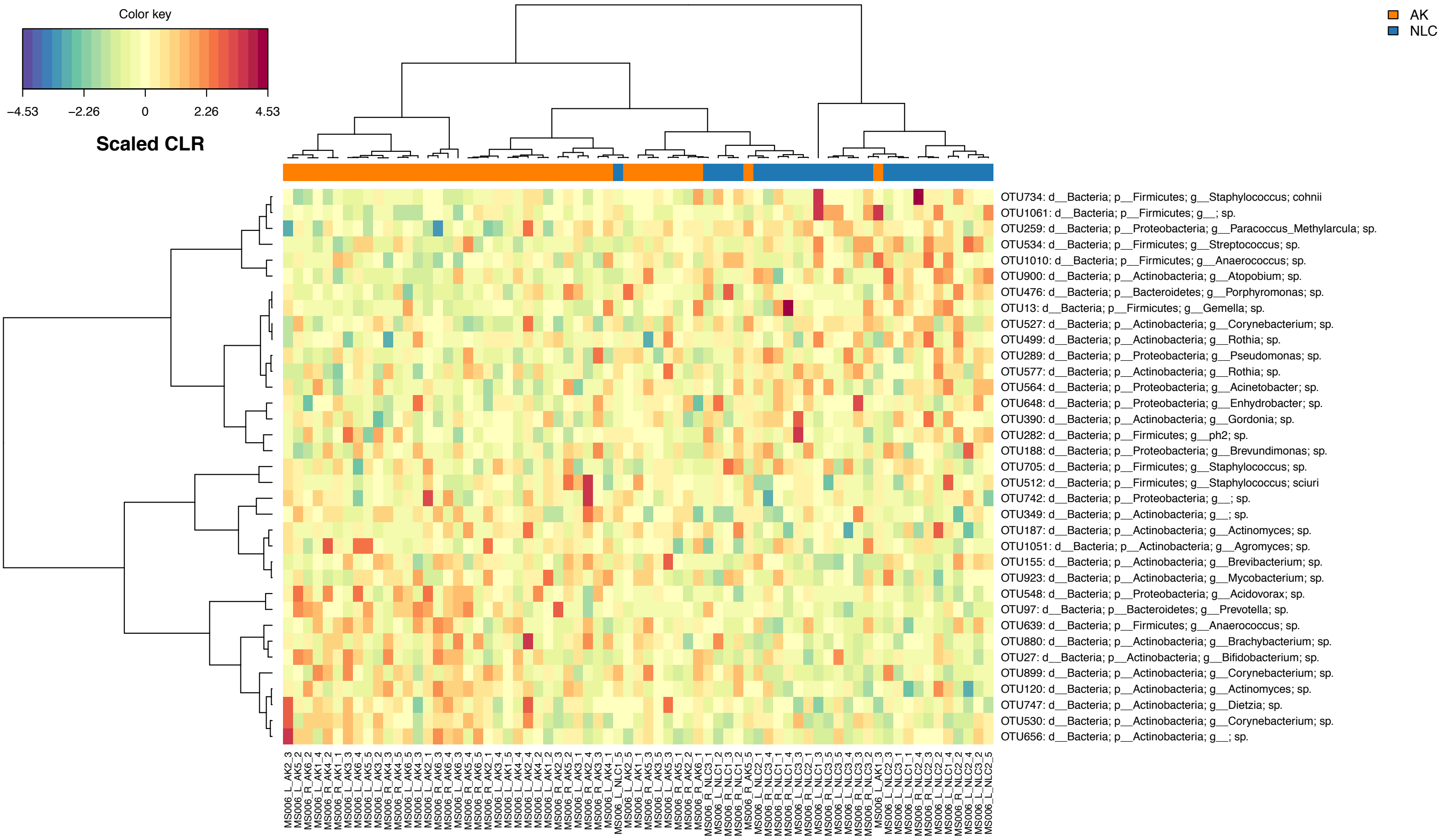

Figure S1l. Multivariate analysis highlight a microbial signature separating AK and NLC samples for subject MS006.

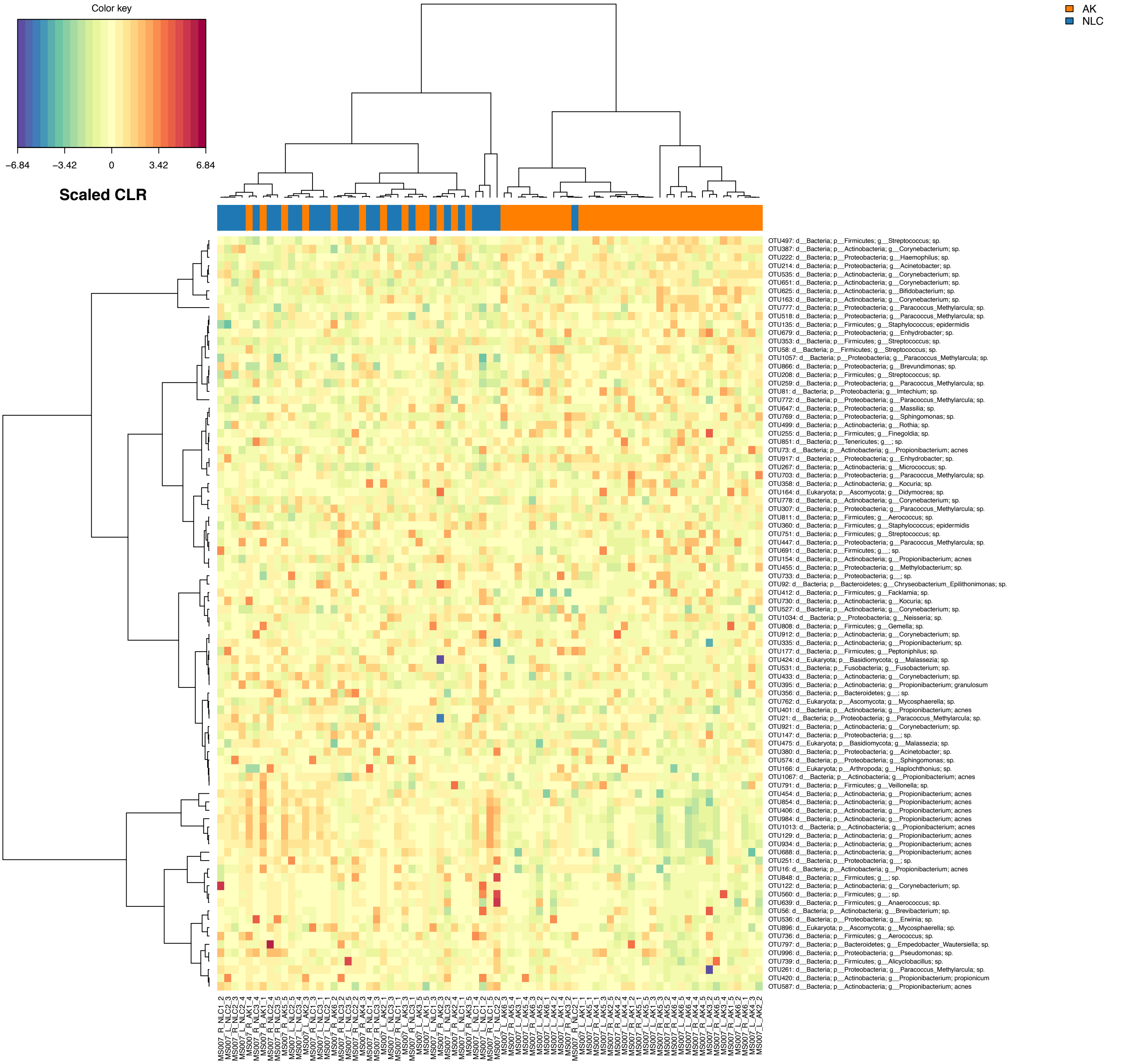

Figure S1m. Multivariate analysis highlight a microbial signature separating AK and NLC samples for subject MS007.

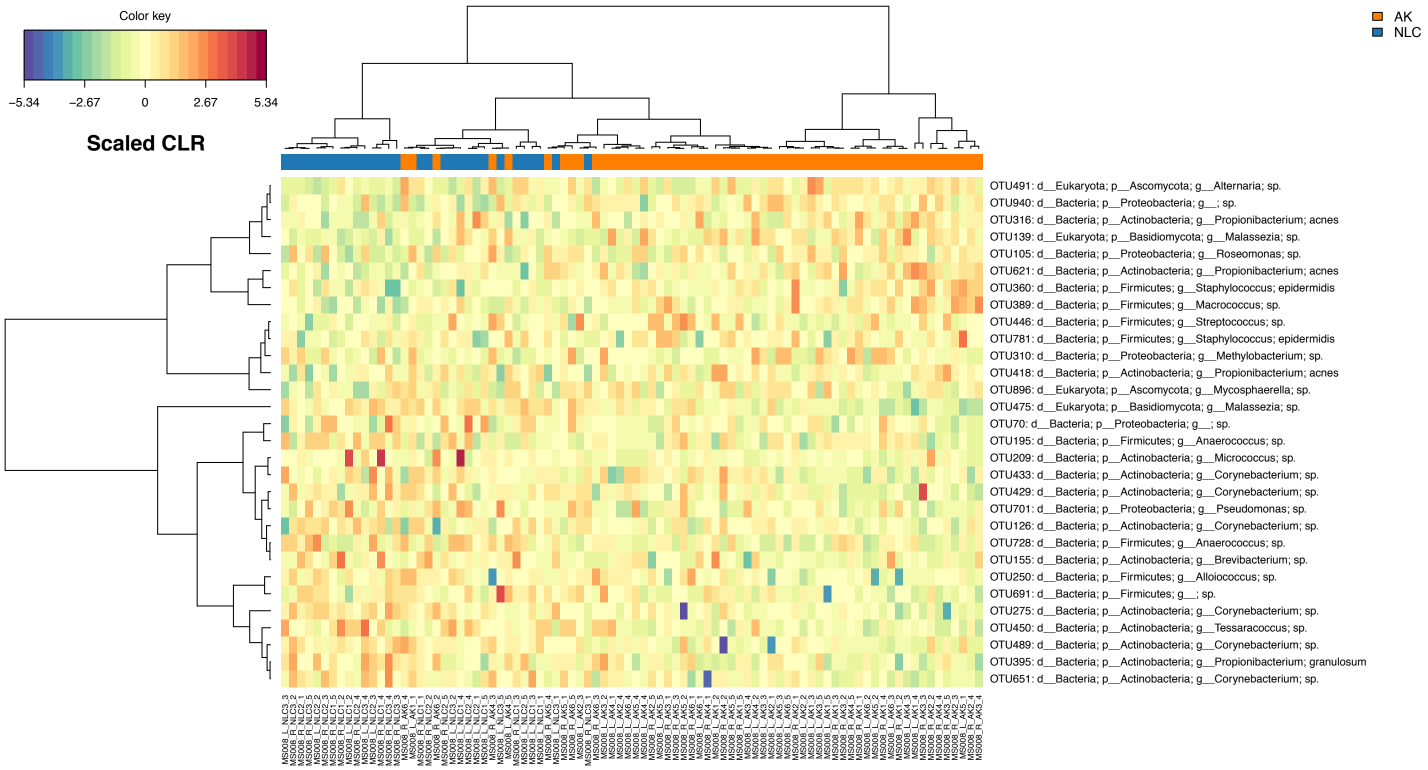

Figure S1n. Multivariate analysis highlight a microbial signature separating AK and NLC samples for subject MS008.

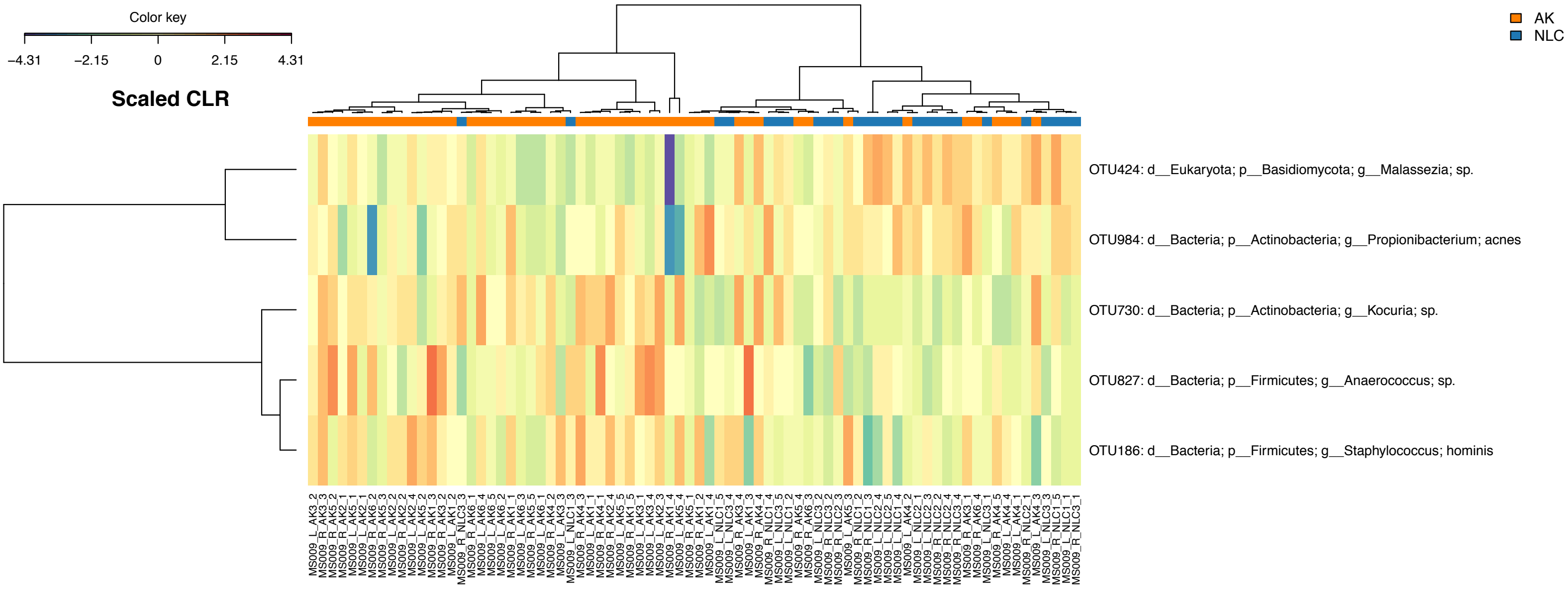

Figure S1o. Multivariate analysis highlight a microbial signature separating AK and NLC samples for subject MS009.

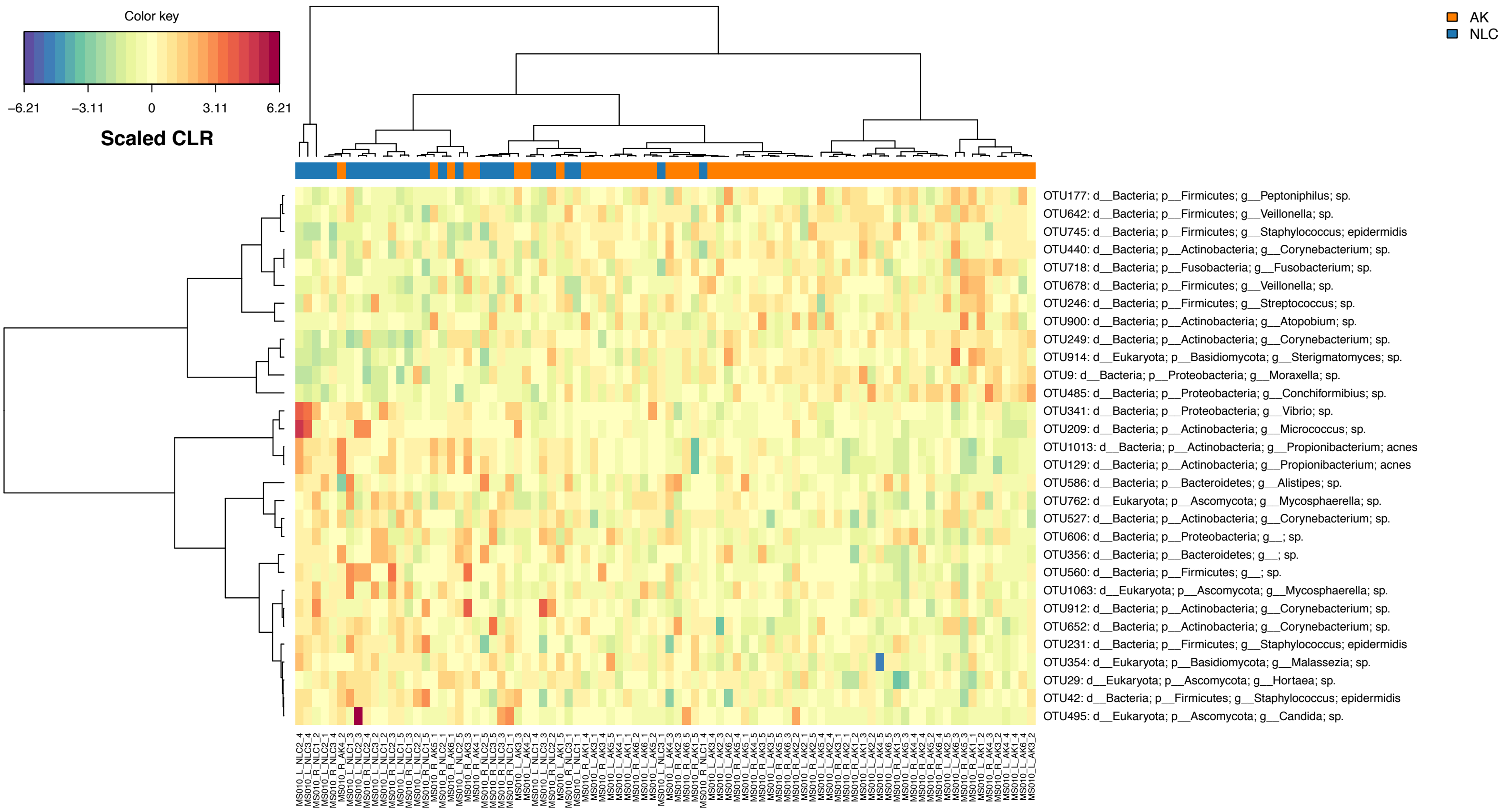

Figure S1p. Multivariate analysis highlight a microbial signature separating AK and NLC samples for subject MS010.

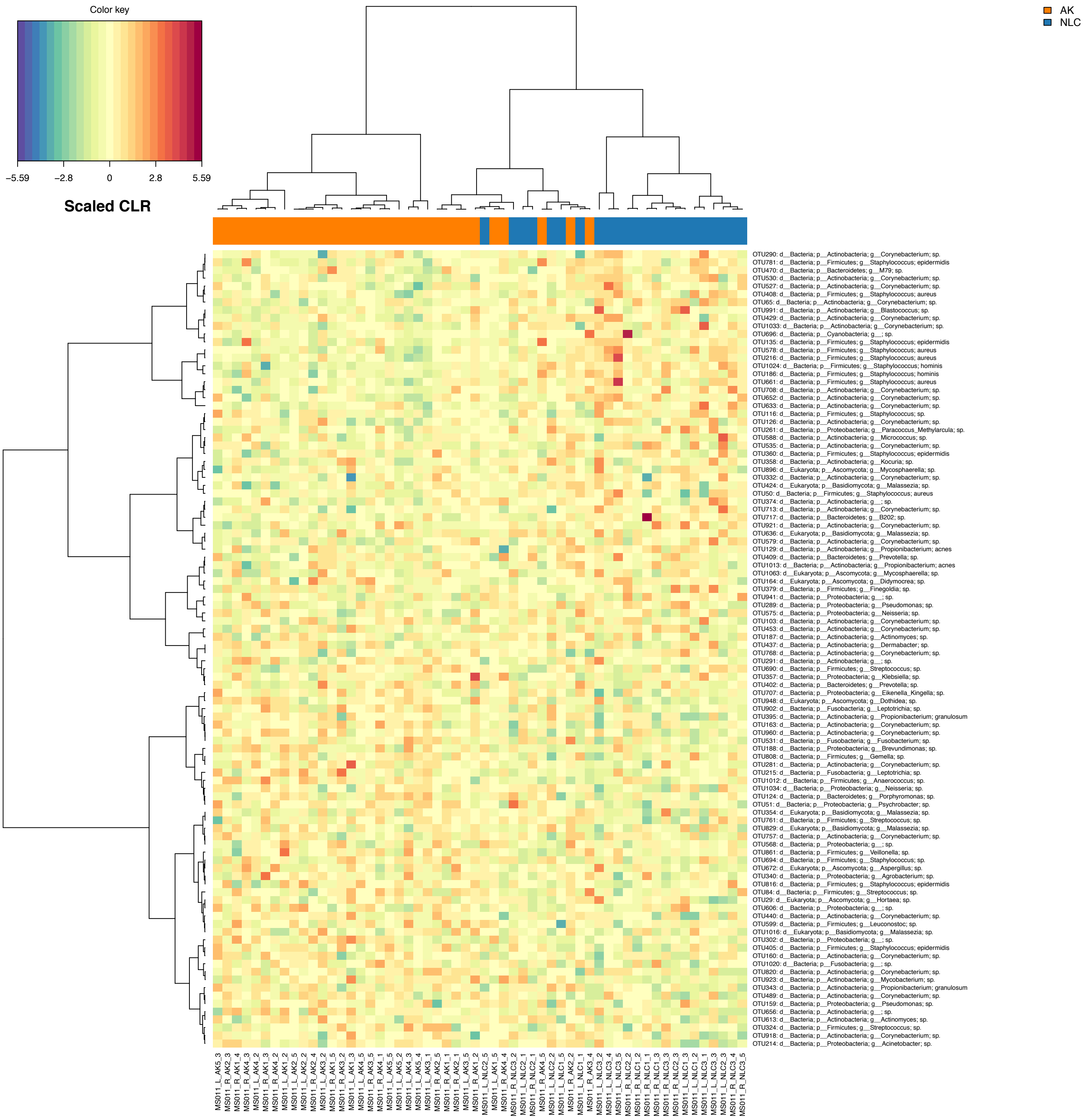

Figure S1q. Multivariate analysis highlight a microbial signature separating AK and NLC samples for subject MS011.

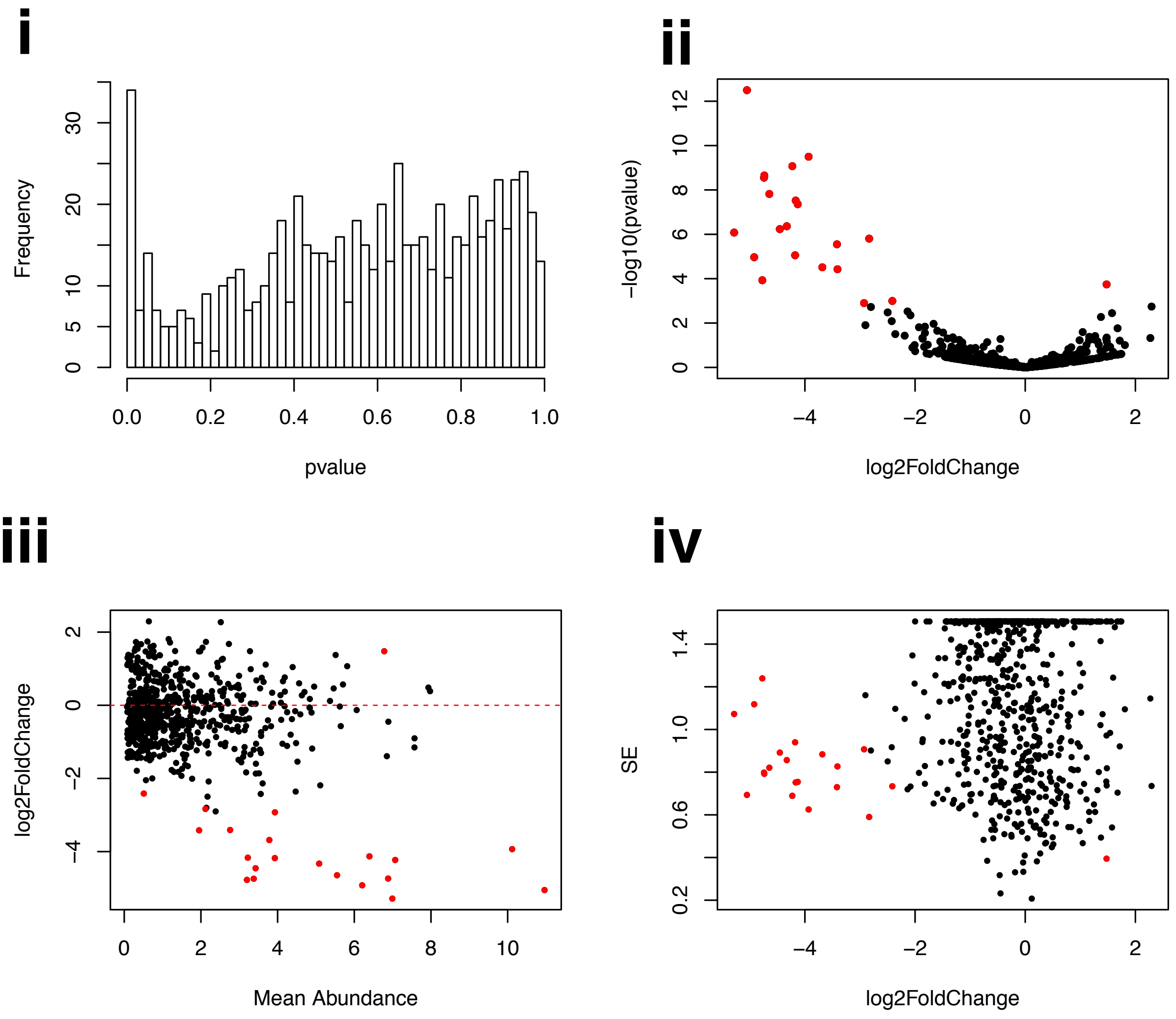

Figure S1r. Differential abundance of SCC and SCC_PL samples. (i) P-value histogram of DESeq2 results. (ii) Volcano plot indicating differentially abundant OTUs in red, and all other testable OTUs in black points. All but one of the differentially abundant OTUs were higher in abundance in SCC than SCC_PL samples. (iii) MA plot showing the log2 fold change against mean abundance of each OTU. (iv) Standard error plot, showing the log2 fold change plotted against the DESeq2 standard error calculation.

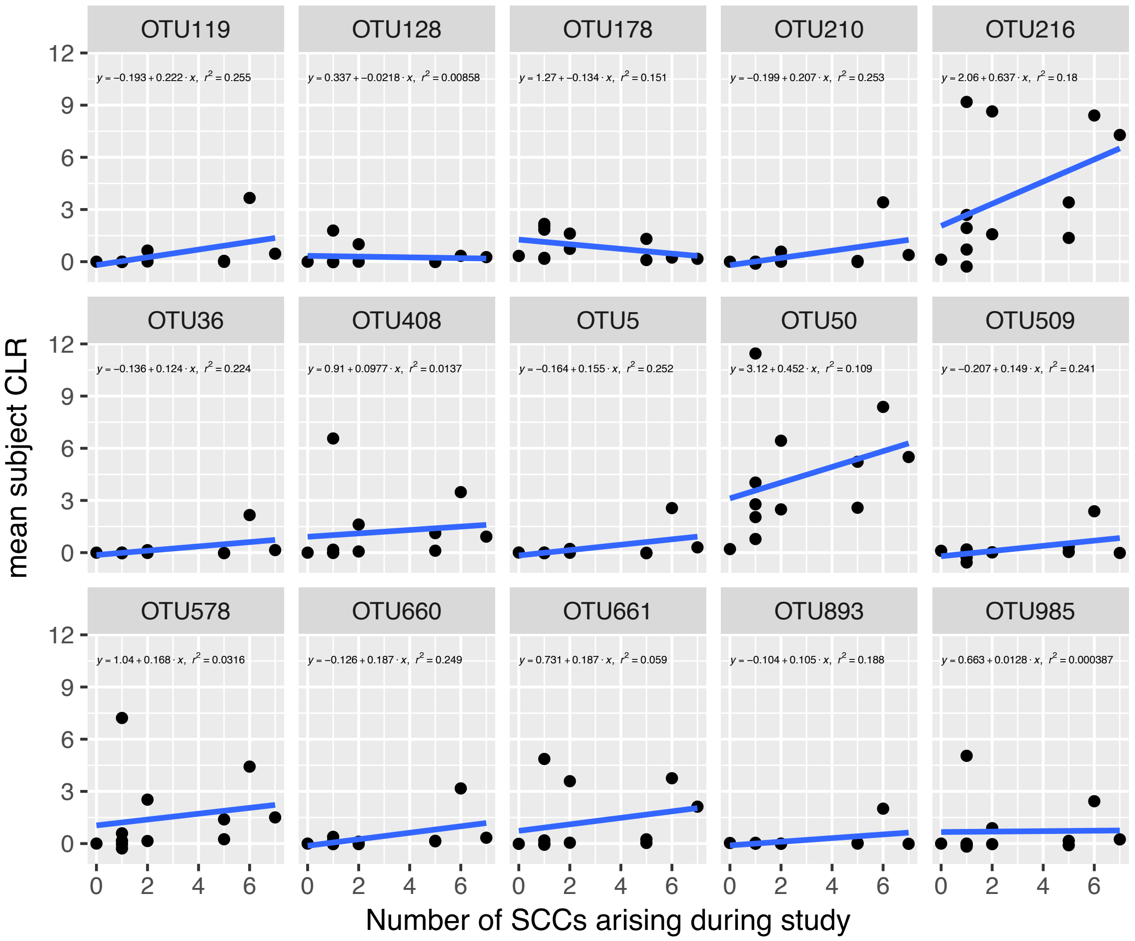

Figure S1s. Correlations between the number of SCCs developing during this study, and OTU abundance for all subjects. A weak positive correlation was observed between OTUs 216 and 50 and the number of SCCs.

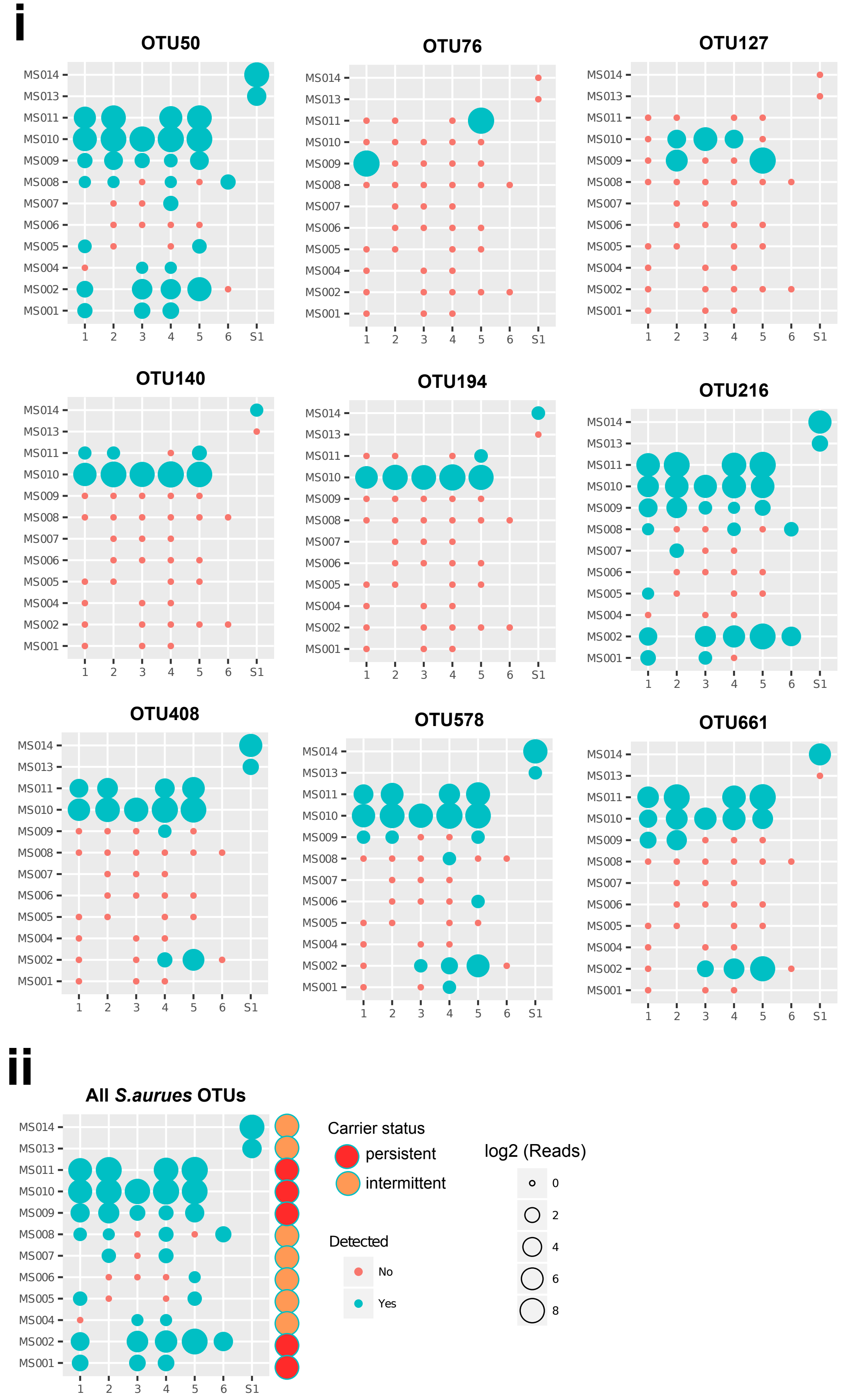

Figure S1t. *Staphylococcus aureus* carrier status. (i) Read counts for all *S. aureus* OTUs in the study for all nasal swab samples. Each row is a participant, and each column a visit time point. Circles represent the log2 (read counts). Red dots indicate that the sample was tested, and no reads were detected. Empty spots indicate no data available. (ii) Cumulative read count for all *S.aureus* OTUs. Persistent carriers (S.aureus detected in every sample), are indicated with a red circle, and intermittent carriers with an orange circle. All subjects carried *S.aureus* in their nasal passages either persistently or intermittently.

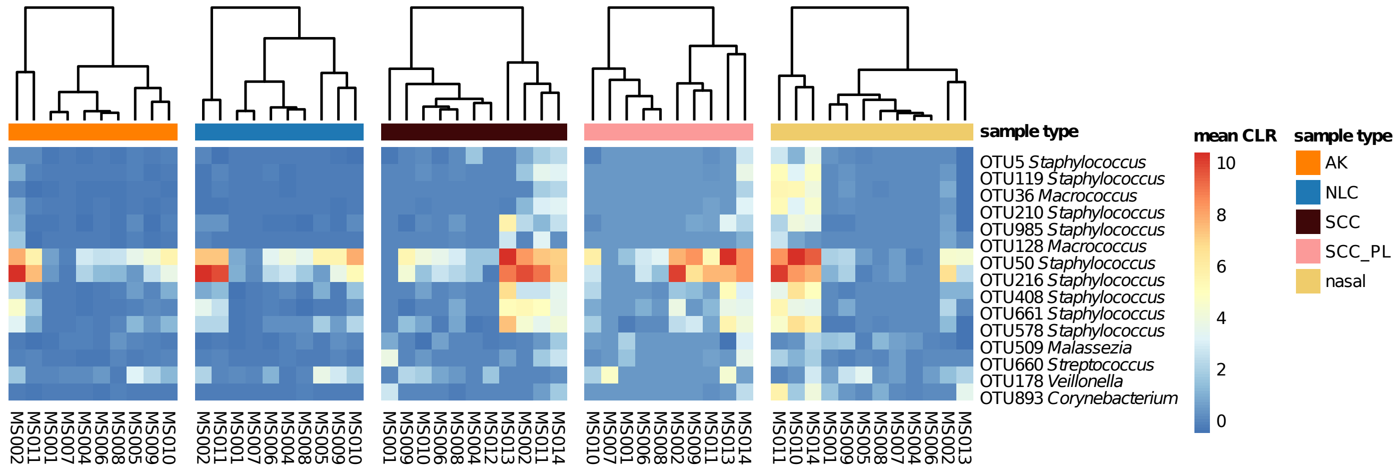

Figure S1u. Nasal profiles and abundance of strongly SCC-associated OTUs in non-lesional skin. Heatmap of mean CLR for all SCC associated OTUs with an importance above 0.1 across each sample type. A subset of subjects carry these OTUs in their nasal passages, in particular OTU 216 and 50. Both of these OTUs are present on the photo-damaged nonlesional control (NLC) skin of most subjects, but high in abundance on subjects MS002, 10, 11, 12, 13 and 14. Most other subjects carry these OTUs but at much lower abundance.

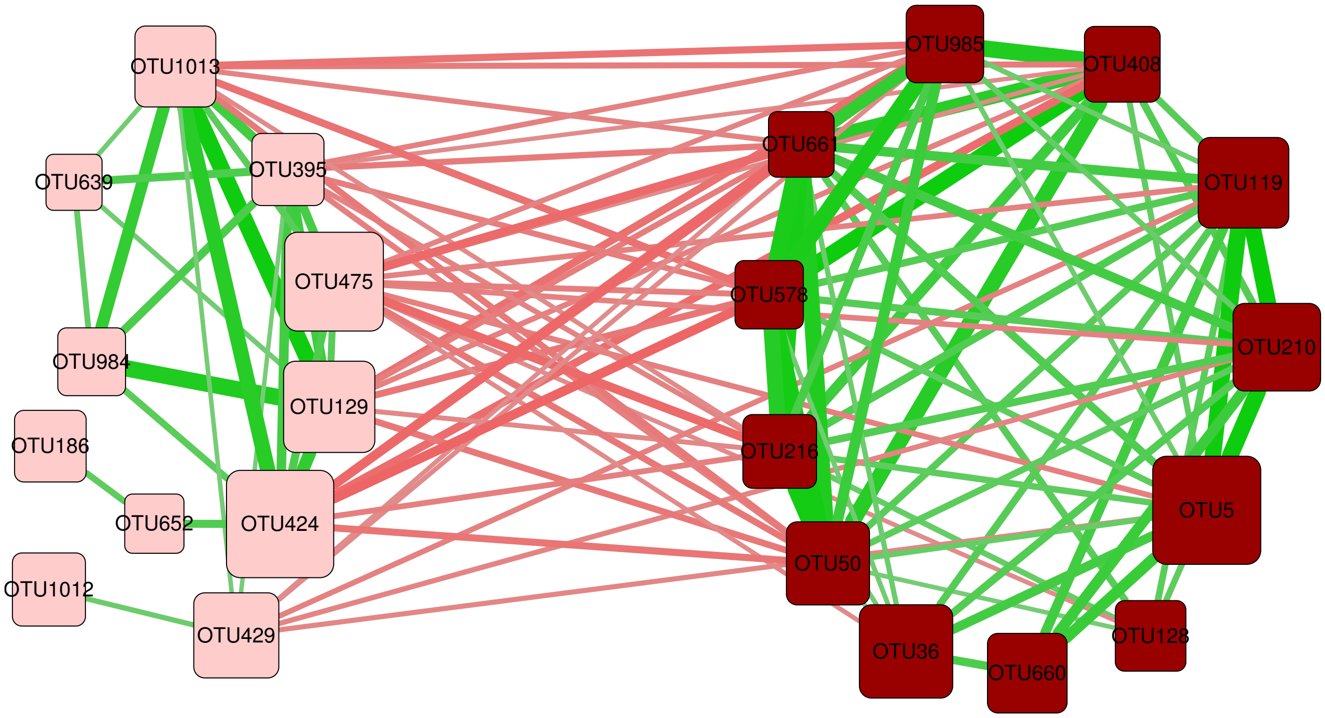

Figure S1v. Abundance correlation network for the SCC and SCC_PL samples. Two highly interconnected modules of positively correlate OTUs (green edges, width is proportional to significance of correlation), one containing *Propionibacterium* and *Malassezia* OTUs (left hand side, light pink OTU colours) is strongly negatively correlated (red edges) in abundance with the SCC-associated module containing predominately *S. aureus* OTUs (right hand side, purple OTUs).
